# Correspondence of Contradictions in the Constructive Connexive Calculus **C**

**DOI:** 10.1007/s00153-026-01013-7

**Published:** 2026-05-02

**Authors:** Satoru Niki

**Affiliations:** 1 Ruhr University Bochum, Department of Philosophy I, Universitätsstraße 150, Bochum, Germany; 2 Department of Applied Systems and Mathematics, Kanagawa University, 3-27-1 Rokkakubashi, Kanagawa-ku, 221-8686 Yokohama, Kanagawa Japan

**Keywords:** connexive logic, constructive logic, negation inconsistency, sequent calculus, tableau calculus, 03B53, 03B60, 03F03, 03F05, 03F07

## Abstract

The calculus **C** was introduced by H. Wansing as a constructive logic with strong negation. In addition, **C** validates the theses of connexive logic that are attributed to Aristotle and Boethius. A further remarkable property of **C** is that it is a non-trivial but negation inconsistent system: it has a formula and its negation as theorems. From a bilateralist-minded perspective, such a contradiction can be seen as the existence of both a verification and a falsification of one and the same formula. Relatedly, it has been noted by Wansing that there seems to be a kind of correspondence between these two types of derivations when it comes to a proof of contradiction. Following this observation, we attempt in this paper to introduce a precise notion for such a correspondence. We thence establish that this correspondence obtains in propositional and first-order versions of **C**, via formulations of suitable sequent and tableau calculi.

## Introduction

The calculus **C**, introduced by H. Wansing [22], is an expansion of positive intuitionistic logic with a negation which exhibits *connexive* features: it validates classically invalid schemata known as *Aristotle’s theses* $\mathord {\sim }(\mathord {\sim }A\rightarrow A)$ and $\mathord {\sim }(A\rightarrow \mathord {\sim }A)$, as well as *Boethius’ theses* $(A\rightarrow B)\rightarrow \mathord {\sim }(A\rightarrow \mathord {\sim }B)$ and $(A\rightarrow \mathord {\sim }B)\rightarrow \mathord {\sim }(A\rightarrow B)$. (see [12, 24, 27] for details.) It can also be seen as a variant of A. Almukdad and D. Nelson’s constructive paraconsistent logic **N4** [1]. The negation of **C** can for this reason be categorised as a *constructible falsity* or a *strong negation* [14]. Proof-theoretically^1^, this means that **C** can be seen as a system which treats the notions of *verification* and *falsification* on a par, so that a sequent can be formulated in the form^2^$\Gamma :\Delta \Rightarrow ^{*}A$, where $*\in \{+,-\}$ stands for these notions, and $\Gamma$ and $\Delta$ are premises on falsified/verified formulas. The negation then serves as a bridge between the two notions, and a provable contradiction can be rephrased as a simultaneous verification and falsification of the same formula.

Interesting aspects of **C** are not limited to connexivity. Another remarkable property of **C** is that it is a (non-trivial) *negation inconsistent* system, in the sense that it has a contradictory pair of a formula and its negation as theorems. The key for the negation inconsistency lies in the treatment of negated implication, since it is the only difference from the negation consistent **N4**. If we employ the aforementioned perspective of sequents, then it is possible (as we shall see) to point out a parallel treatment of the verification and falsification of an implication. Heinrich Wansing pointed out to the author (in a private communication) that a generalisation of such a correspondence can be found in derivations of a contradiction: His example concerns the formula $\mathord {\sim }A\rightarrow \mathord {\sim }(A\rightarrow \mathord {\sim }A)$. This formula can be both refuted and verified by the next pair of derivations: 
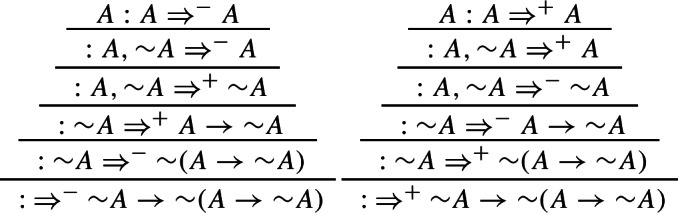


where the fourth and the last lines exhibit parallel rules for introducing implication, and the second, third and fifth lines exhibit parallel rules for introducing negation.

The question we ask in this paper is to what extent this example can be generalised. The aim therefore is to spell out the correspondence between verification and falsification implicit in the above example, and to establish that such a correspondence is obtainable for any given instance of provable contradictions in **C**. Achieving this aim leads to a better understanding of the negation inconsistency of **C**, as it shows a necessary structural relationship between the notions of verification and falsification for provable contradictions. Furthermore, due to the dependence of the enquiry on specific proof systems, a characterisation may also give an insight into the question of which proof system is conceptually to be preferred.

This paper is structured as follows: in Section 2, we shall introduce the propositional sequent calculus used in the above example, and show properties that are necessary for our investigation. In addition, we recall the Kripke semantics for **C** introduced in [22] which will be used in some places. Section 3 first defines the notions of correspondence. Then as an initial step we shall investigate the implication-negation fragment of **C**, and show how a pair of derivations satisfying the correspondence can be constructed from a given instance of provable contradictions. This is followed by an analysis in terms of propositional variables that offers a necessary condition all derivations of a provable contradiction must satisfy. In Section 4, we shall attempt to generalise the results of the previous section in the full propositional calculus, the initial outcome of which is a correspondence under a restriction on formulas. We will then suggest a way to remove this restriction by transforming the sequent calculus to a Mints-style [13] tableau calculus. Finally, Section 5 and 6 develop the analysis for the first-order version of **C** by slightly altering the definitions of correspondence.

## Preliminaries for the propositional case

In this section, we shall introduce target propositional logics of our enquiry via proof systems and semantics.

### Proof systems

Let $p_{1},p_{2},\ldots$ be a countably infinite supply of *propositional variables*. We shall use $p,q,\ldots$ and $A,B,\ldots$ as metavariables for propositional variables and formulas, respectively. Formulas in two languages $\mathcal {L}_{\rightarrow }$ and $\mathcal {L}$ are specified in the next way.

$$\begin{aligned} (\mathcal {L}_{\rightarrow }) A  &   {::}{=} p\ |\ (A\rightarrow A)\ |\ \mathord {\sim }A.\\ (\mathcal {L}) A  &   {::}{=} p\ |\ (A\wedge A)\ |\ (A\vee A)\ |\ (A\rightarrow A)\ |\ \mathord {\sim }A. \end{aligned}$$

In addition, we shall use abbreviations $(A\leftrightarrow B)$ and $\top$ for $(A\rightarrow B)\wedge (B\rightarrow A)$ and $p_{0}\rightarrow p_{0}$ for a specific propositional variable $p_{0}$.

The *complexity* |*A*| for a formula *A* is given by the next clauses: $|p|=0$, $|A\circ B|=|A|+|B|+2$ ($\circ \in \{\wedge ,\vee ,\rightarrow \}$) and $|\mathord {\sim }A|=|A|+1$.

#### Definition 1

The propositional logic **C** is defined as a Hilbert-style system with the following set of axiom schemata and the rule of *modus ponens*.

A1 $$\begin{aligned} (A{\rightarrow }(B{\rightarrow }C)){\rightarrow }((A{\rightarrow }B){\rightarrow }(A{\rightarrow }C)) \end{aligned}$$

A2 $$\begin{aligned} A\rightarrow (B\rightarrow A) \end{aligned}$$

A3 $$\begin{aligned} (A\rightarrow \mathord {\sim }B)\rightarrow \mathord {\sim }(A\rightarrow B) \end{aligned}$$

A4 $$\begin{aligned} \mathord {\sim }(A\rightarrow B)\rightarrow (A\rightarrow \mathord {\sim }B) \end{aligned}$$

A5 $$\begin{aligned} A\rightarrow \mathord {\sim }\mathord {\sim }A \end{aligned}$$

A6 $$\begin{aligned} \mathord {\sim }\mathord {\sim }A\rightarrow A \end{aligned}$$

A7 $$\begin{aligned} A\rightarrow (B\rightarrow (A\wedge B)) \end{aligned}$$

A8 $$\begin{aligned} (A_{1}\wedge A_{2})\rightarrow A_{i} \end{aligned}$$

A9 $$\begin{aligned} A_{i}\rightarrow (A_{1}\vee A_{2}) \end{aligned}$$

A10 $$\begin{aligned} (A{\rightarrow }C){\rightarrow }((B{\rightarrow }C){\rightarrow }((A{\vee }B){\rightarrow }C)) \end{aligned}$$

A11 $$\begin{aligned} \mathord {\sim }(A\wedge B)\leftrightarrow (\mathord {\sim }A\vee \mathord {\sim }B) \end{aligned}$$

A12 $$\begin{aligned} \mathord {\sim }(A\vee B)\leftrightarrow (\mathord {\sim }A\wedge \mathord {\sim }B) \end{aligned}$$

MP $$\begin{aligned} {\frac{A\qquad A\rightarrow B}{B}} \end{aligned}$$

where $i\in \{1,2\}$. The implication-negation system ${\textbf {C}}_{\rightarrow }$ in $\mathcal {L}_{\rightarrow }$ is then defined from (A1)–(A6) and (MP). In each case, a derivation of a formula *A* from a set of formulas $\Gamma$ is a finite sequence of formulas $B_{1},\ldots ,B_{n}\equiv A$, where each $B_{i}$ is either an instance of an axiom schema, an element of $\Gamma$, or obtained from preceding formulas by (MP).

For our enquiry, we shall mainly concern ourselves with another type of proof system, namely sequent calculus. Following the presentation of Wansing’s example, the system we shall use is a simple modification of bilateral^3^ sequent calculi **Sn4** and **Dn4** introduced by N. Kamide and Wansing in [9, 10] for **N4**. More specifically, a *sequent* will be of the form $\Gamma :\Delta \Rightarrow ^{*}A$, where $\Gamma$ and $\Delta$ are finite multisets of formulas called *negative/positive antecedent*, and $*\in \{+,-\}$ is the *sign* of the consequent formula. This framework is suitable for our purpose in simplifying definitions and making explicit the role of the polarity that is kept implicit in the Hilbert-style calculi.

When it comes to structural rules, we shall use a **G3**-type formulation [15, 21] which absorbs them. Some other rules are defined like in **GK**-type systems [21], in that their principal formula (the displayed formula in the conclusion) is duplicated in the premises.^4^

#### Definition 2

The sequent calculus $\textbf{bGKC}$ is defined by the following set of rules:
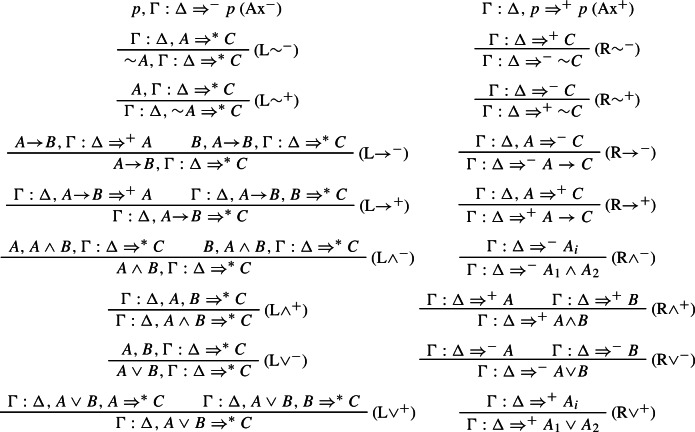


where $*\in \{+,-\}$ and $i\in \{1,2\}$. The implication-negation system $\textbf{bGKC}_{\rightarrow }$ is then defined in $\mathcal {L}_{\rightarrow }$ by removing the rules for conjunction and disjunction. A *derivation* of a sequent is a finite tree which has the sequent as the root, and whose leaves (*initial sequents*) are instances of ($\text {Ax}^-$) or ($\text {Ax}^+$), and the sequent in each non-leaf node is obtained by one of the rules from sequent(s) of the preceding nodes.

Given a derivation in a sequent calculus, its *depth* is the number of non-leaf nodes in the longest branch of the tree (it can be defined for a derivation in a Hilbert-style system by considering it as a tree).

Both ($\text {Ax}^-$) and ($\text {Ax}^+$) can be generalised into all formulas.

#### Proposition 1

The following statements hold.

(i) $${\textbf {bGKC}}_{\rightarrow }\vdash A,\Gamma :\Delta \Rightarrow ^{-} A$$ and $${\textbf {bGKC}}_{\rightarrow }\vdash \Gamma :\Delta ,A\Rightarrow ^{+} A$$.
(ii) $$\textbf{bGKC}\vdash A,\Gamma :\Delta \Rightarrow ^{-} A$$ and $$\textbf{bGKC}\vdash \Gamma :\Delta ,A\Rightarrow ^{+} A$$.

#### Proof

By induction on the complexity of *A*. $\square$

### Cut-elimination

One desirable property for a sequent calculus is cut-elimination. For **bGKC**, this can be shown in a standard manner (see e.g. [15, 21]), with a small difference that we have two cut rules, for $\Rightarrow ^{+}$ and $\Rightarrow ^{-}$ (cf. the cases for **Sn4** and **Dn4** [9, 10]).

Let us use $\vdash _{k}$ to denote the derivability of a sequent with depth at most *k*. We shall say a rule is *depth-preserving admissible* if the existence of derivations of its premises implies the existence of a derivation of its conclusion with depth at most the maximum depth of the derivations of the premises. When there is no restriction about the depth, we shall say a rule is *admissible*.

We begin with observing the depth-preserving admissibility of *weakening* rules.

#### Lemma 2

The next rules are depth-preserving admissible in ${\textbf {bGKC}}_{\rightarrow }$ and $\textbf{bGKC}$.

$$\begin{aligned} \frac{\Gamma :\Delta \Rightarrow ^{*}C}{A,\Gamma :\Delta \Rightarrow ^{*}C} {\textit{(}LW^-\textit{)}} \frac{\Gamma :\Delta \Rightarrow ^{*}C}{\Gamma :\Delta ,A\Rightarrow ^{*}C} {\textit{(}LW^+\textit{)}} \end{aligned}$$

#### Proof

By induction on the depth of derivations. $\square$

The weakening rules can be used to show that most^5^ of the rules in ${\textbf {bGKC}}_{\rightarrow }$ and $\textbf{bGKC}$ are (depth-preserving) *invertible*, i.e. the existence of a derivation of the conclusion guarantees the existence of a derivation of the premise without an increase in depth.

Specifically, we have the next lemma.

#### Lemma 3

The following statements hold in ${\textbf {bGKC}}_{\rightarrow }$ and $\textbf{bGKC}$.

(i) If $$\vdash _{k}\mathord {\sim }A,\Gamma :\Delta \Rightarrow ^{*}C$$ then $$\vdash _{k}\Gamma :\Delta ,A\Rightarrow ^{*}C$$.
(ii) If $\vdash _{k}\Gamma :\Delta ,\mathord {\sim }A\Rightarrow ^{*}C$ then $\vdash _{k}A,\Gamma :\Delta \Rightarrow ^{*}C$.
(iii) If $\vdash _{k}\Gamma :\Delta \Rightarrow ^{-}\mathord {\sim }C$ then $\vdash _{k}\Gamma :\Delta \Rightarrow ^{+}C$.
(iv) If $\vdash _{k}\Gamma :\Delta \Rightarrow ^{+}\mathord {\sim }C$ then $\vdash _{k}\Gamma :\Delta \Rightarrow ^{-}C$.
(v) If $$\vdash _{k}A\rightarrow B,\Gamma :\Delta \Rightarrow ^{*} C$$ then $$\vdash _{k}B, A\rightarrow B,\Gamma :\Delta \Rightarrow ^{*} C$$.
(vi) If $\vdash _{k}\Gamma :\Delta , A\rightarrow B \Rightarrow ^{*} C$ then $\vdash _{k}\Gamma :\Delta ,A\rightarrow B, B \Rightarrow ^{*} C$.
(vii) If $\vdash _{k}\Gamma :\Delta \Rightarrow ^{-} A\rightarrow B$ then $\vdash _{k}\Gamma :\Delta , A \Rightarrow ^{-} B$.
(viii) If $\vdash _{k}\Gamma :\Delta \Rightarrow ^{+} A\rightarrow B$ then $\vdash _{k}\Gamma :\Delta , A \Rightarrow ^{+} B$.

Moreover, the next statements hold in $\textbf{bGKC}$ (where $i\in \{1,2\}$).

(ix) If $\vdash _{k} A_{1}\wedge A_{2},\Gamma :\Delta \Rightarrow ^{*}C$ then $\vdash _{k}A_{i},A_{1}\wedge A_{2},\Gamma :\Delta \Rightarrow ^{*}C$.
(x) If $$\vdash _{k} \Gamma :\Delta ,A\wedge B\Rightarrow ^{*}C$$ then $$\vdash _{k}\Gamma :\Delta ,A,B\Rightarrow ^{*}C$$.
(xi) If $\vdash _{k} \Gamma :\Delta \Rightarrow ^{+}A_{1}\wedge A_{2}$ then $\vdash _{k}\Gamma :\Delta \Rightarrow ^{+}A_{i}$.
(xii) If $\vdash _{k} A\vee B,\Gamma :\Delta \Rightarrow ^{*}C$ then $\vdash _{k}A,B,\Gamma :\Delta \Rightarrow ^{*}C$.
(xiii) If $\vdash _{k}\Gamma :\Delta \Rightarrow ^{-}A_{1}\vee A_{2}$ then $\vdash _{k}\Gamma :\Delta \Rightarrow ^{-}A_{i}$.
(xiv) If $\vdash _{k}\Gamma :\Delta ,A_{1}\vee A_{2}\Rightarrow ^{*}C$ then $\vdash _{k}\Gamma :\Delta ,A_{1}\vee A_{2},A_{i}\Rightarrow ^{*}C$.

#### Proof

By induction on the depth of derivations, with Lemma 2 for (v)–(ix), (xiv). $\square$

This in turn implies the depth-preserving admissibility of *contraction* rules.

#### Lemma 4

The next rules are depth-preserving admissible in ${\textbf {bGKC}}_{\rightarrow }$ and $\textbf{bGKC}$.

$$\begin{aligned} \frac{A,A,\Gamma :\Delta \Rightarrow ^{*}C}{A,\Gamma :\Delta \Rightarrow ^{*}C} {\textit{(}LC^-\textit{)}} \frac{\Gamma :\Delta ,A,A\Rightarrow ^{*}C}{\Gamma :\Delta ,A\Rightarrow ^{*}C} {\textit{(}LC^+\textit{)}} \end{aligned}$$

#### Proof

The cases follow from inductions on the depth of derivations. The cases for (L$\wedge ^-$) and (L$\vee ^+$) use Lemma 3. $\square$

Contraction guarantees more **G3**-style inversions of some of the left rules.

#### Lemma 5

The following statements hold in ${\textbf {bGKC}}_{\rightarrow }$ and $\textbf{bGKC}$.

(i) If $$\vdash _{k}A\rightarrow B,\Gamma :\Delta \Rightarrow ^{*} C$$ then $$\vdash _{k}B,\Gamma :\Delta \Rightarrow ^{*} C$$.
(ii) If $\vdash _{k}\Gamma :\Delta , A\rightarrow B \Rightarrow ^{*} C$ then $\vdash _{k}\Gamma :\Delta , B \Rightarrow ^{*} C$.

Moreover, the next statements hold in $\textbf{bGKC}$ (where $i\in \{1,2\}$).

(iii) If $\vdash _{k} A_{1}\wedge A_{2},\Gamma :\Delta \Rightarrow ^{*}C$ then $\vdash _{k}A_{i},\Gamma :\Delta \Rightarrow ^{*}C$.
(iv) If $\vdash _{k}\Gamma :\Delta ,A_{1}\vee A_{2}\Rightarrow ^{*}C$ then $\vdash _{k}\Gamma :\Delta ,A_{i}\Rightarrow ^{*}C$.

#### Proof

By induction on the depth of derivations, using Lemma 4. $\square$

We are now ready to show the admissibility of cut in **bGKC** and ${\textbf {bGKC}}_{\rightarrow }$.

#### Theorem 6

The following rules are admissible in $\textbf{bGKC}$ and ${\textbf {bGKC}}_{\rightarrow }$.

$$\begin{aligned} \frac{\Gamma :\Delta \Rightarrow ^{-} A \qquad A,\Gamma ':\Delta '\Rightarrow ^{*}C}{\Gamma ,\Gamma ':\Delta ,\Delta '\Rightarrow ^{*}C} {\textit{(}Cut^-\textit{)}} \frac{\Gamma :\Delta \Rightarrow ^{+} A \qquad \Gamma ':\Delta ',A\Rightarrow ^{*}C}{\Gamma ,\Gamma ':\Delta ,\Delta '\Rightarrow ^{*}C} {\textit{(}Cut^+\textit{)}} \end{aligned}$$

#### Proof

Let us call the formula represented above by *A* the *cutformula*. The statement will be shown by induction on the complexity of the cutformula, with a subinduction on the *level* of cut (sum of the depths of the subderivations of the premises). The cases for ($\text {Cut}^-$) and ($\text {Cut}^+$) are simultaneously treated. Here we look at two cases for ($\text {Cut}^-$) where the cutformula is principal in both of the premises.

If the cutformula has the form $\mathord {\sim }B$, then we have the next instance: 
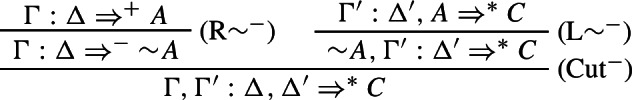
 Then we can construct the next derivation:

$$\begin{aligned} \frac{\Gamma :\Delta \Rightarrow ^{+}A \qquad \Gamma ':\Delta ',A\Rightarrow ^{*}C}{\Gamma ,\Gamma ':\Delta ,\Delta '\Rightarrow ^{*}C} {(\text {Cut}^+)} \end{aligned}$$

where the ($\text {Cut}^+$) is of less complexity, and so admissible by the I.H..

If the cutformula has the from $B\rightarrow C$, then we have the next instance for ($\text {Cut}^-$): 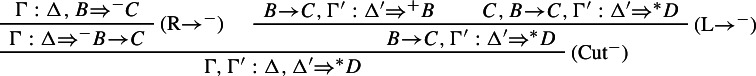 Then on one hand we can derive: 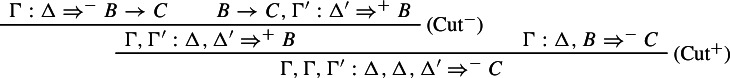 where the upper ($\text {Cut}^-$) is admissible as it has less level, while the lower ($\text {Cut}^+$) is admissible as it has less complexity. The derivation is continued as follows, utilising again the admissibility of ($\text {Cut}^-$) by the I.H. along with that of ($\text {LC}^-$) and ($\text {LC}^+$).
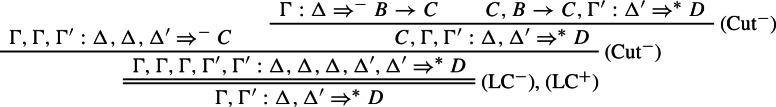
$$\square $$

As a consequence of Theorem 6, we can establish the correspondence between the Hilbert-style systems and the sequent calculi. Let us define $A^{-}$ and $A^{+}$ to be $\mathord {\sim }A$ and *A* itself, respectively. Then $\Gamma ^{-}$ denotes the multiset $\{A^{-}:A\in \Gamma \}$. Moreover, we adopt the convention that $\bigwedge \Gamma \equiv \top$ if $\Gamma =\emptyset$. Finally, if $B_{1},\ldots , B_{n}$ are the elements of $\Gamma$, then $\Gamma \rightarrow A$ stands for $B_{1}\rightarrow (B_{2}\rightarrow (\ldots \rightarrow (B_{n}\rightarrow A))$.

#### Corollary 7

The following statements hold.

(i) ${\textbf {bGKC}}_{\rightarrow }\vdash \Gamma :\Delta \Rightarrow ^{*}A$ if and only if ${\textbf {C}}_{\rightarrow }\vdash \Gamma ^{-}\rightarrow (\Delta \rightarrow A^{*})$.
(ii) $\textbf{bGKC}\vdash \Gamma :\Delta \Rightarrow ^{*}A$ if and only if $\textbf{C}\vdash (\bigwedge \Gamma ^{-}\wedge \bigwedge \Delta )\rightarrow A^{*}$.

#### Proof

(i) The right-to-left direction follows from showing that the derivability of *A* in the Hilbert-style systems implies the derivability of $\ :\ \Rightarrow ^{+}A$ in the sequent calculi, by induction on the depth of derivations in ${\textbf {C}}_{\rightarrow }$. The statements then follow as a particular case, with the the help of ($\text {Cut}^-$) and ($\text {Cut}^+$). The left-to-right directions follow from induction on the depth of derivations in ${\textbf {bGKC}}_{\rightarrow }$. $\square$

### Semantics

We state in this subsection the Kripke semantics for **C** introduced by Wansing in [22], to which we shall appeal on a few occasions later.

#### Definition 3

A *Kripke frame* $\mathcal {F}$ for **C** is a pair $(W,\le )$ where *W* is a non-empty pre-ordered set, and a *Kripke model* is a triple $(\mathcal {F},\mathcal {V}^{+},\mathcal {V}^{-})$ such that $\mathcal {V}^{+}$ and $\mathcal {V}^{-}$ respectively assigns an upward closed set $\mathcal {V}^{+}(p)$ and $\mathcal {V}^{-}(p)$ for each propositional variable *p*: i.e. if $w\in \mathcal {V}^{*}(p)$ and $w'\ge w$ then $w'\in \mathcal {V}^{*}(p)$ for $*\in \{+,-\}$. They are extended to two forcing relations $\Vdash ^{+}$ and $\Vdash ^{-}$ for all formulas in the next manner.
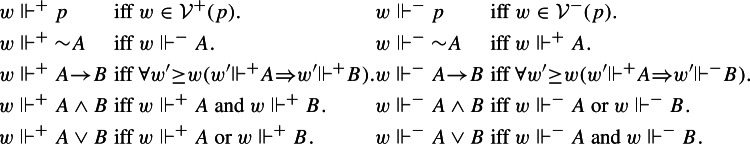


We write $\vDash A$ if $\mathcal {M},w\Vdash ^{+}A$ for all *w* in any model $\mathcal {M}$. The model part will not be denoted explicitly only when it is clear from the context.

A Kripke model for $\textbf{C}_{\rightarrow }$ is obtained by suitably restricting the above model; we shall occasionally write the logic explicitly on the left of ‘$\vDash$’.

#### Theorem 8

The following statements hold.

(i) ${\textbf {bGKC}}_{\rightarrow }\vdash \ :\ \Rightarrow ^{+}A$ if and only if ${\textbf {C}}_{\rightarrow }\vDash A$.
(ii) $\textbf{bGKC}\vdash \ :\ \Rightarrow ^{+}A$ if and only if $\textbf{C}\vDash A$.

#### Proof

By Corollary 7, it suffices to show the completeness with respect to ${\textbf {C}}_{\rightarrow }$ and $\textbf{C}$ in each case. For (ii), see [22]. Then (i) follows from the conservativity of **bGKC** over ${\textbf {bGKC}}_{\rightarrow }$ observable by inspection of the rules. $\square$

## Provable Contradictions in ${\textbf {bGKC}}_{\rightarrow }$

In this section, we shall establish some properties of derivations of provable contradictions in ${\textbf {bGKC}}_{\rightarrow }$, which illustrate how the notion of verification relates to the notion of falsification in ${\textbf {C}}_{\rightarrow }$. To be more precise, by a *provable contradiction*, we shall mean a formula *A* such that both the sequents $\ :\ \Rightarrow ^{+} A$ and $\ :\ \Rightarrow ^{-} A$ are derivable^6^. By (R$\mathord {\sim }^{+}$) and Lemma 3 (iv), this is equivalent to saying that both $\ :\ \Rightarrow ^{+} A$ and $\ :\ \Rightarrow ^{+} \mathord {\sim }A$ are derivable.

### Correspondence of derivations

Let us begin by formalising the correspondence seen in the example in the introduction.

#### Definition 4

We define the notion of *pseudo-derivation* (in ${\textbf {bGKC}}_{\rightarrow }$) from that of derivation by allowing one initial sequent in it to be not an instance of ($\text {Ax}^-$) or ($\text {Ax}^+$), as long as the sequent is derivable in ${\textbf {bGKC}}_{\rightarrow }$.

#### Definition 5

We shall say two sequents *s* and $s'$ *correspond* if $s'$ is obtained from *s* by alternating the sign. Then we shall say two finite sequences $s_{1},\ldots ,s_{n}$ and $s'_{1},\ldots ,s'_{n}$ of sequents *correspond* if $s_{i}$ and $s'_{i}$ correspond for each $1\le i\le n$. Finally, two (pseudo-) derivations *d* and $d'$ (in ${\textbf {bGKC}}_{\rightarrow }$) are said to *correspond* if there are branches *b* in *d* and $b'$ in $d'$ which correspond as finite sequences.

Our first observation is that provable contradictions in ${\textbf {bGKC}}_{\rightarrow }$ always accompany derivations corresponding to each other. This demonstrates a high degree of parity the signs in ${\textbf {bGKC}}_{\rightarrow }$ enjoy. Given the bilateralist interpretation of the signs, this necessity of correspondence for a provable contradiction then suggests that ${\textbf {C}}_{\rightarrow }$ is negation inconsistent because its negation reflects notions which are sufficiently on a par.

#### Theorem 9

If ${\textbf {bGKC}}_{\rightarrow }\vdash \ :\ \Rightarrow ^{+} A$ and ${\textbf {bGKC}}_{\rightarrow }\vdash \ :\ \Rightarrow ^{-} A$, then there are derivations of the sequents which correspond to each other.

#### Proof

Let $d_{a}$ be a derivation of $\ :\ \Rightarrow ^{+} A$ $(=s_{0})$^7^, with $s_{0},\ldots ,s_{n}$ as the rightmost branch. We first construct a pseudo-derivation of $\ :\ \Rightarrow ^{-} A$ that corresponds to $d_{a}$, but its rightmost initial sequent does not have to be an instance of ($\text {Ax}^-$) or ($\text {Ax}^+$). More concretely, we shall show for each $0\le i\le n$, there is a pseudo-derivation $d'_{i}$ corresponding to the pseudo-derivation $d_{i}$ obtained from $d_{a}$ by eliminating the nodes above $s_{i}$.

When $i=0$, the pseudo-derivation $d_{0}$ consists of a single node $\ :\ \Rightarrow ^{+} A$. It is easy to check that a single node $\ :\ \Rightarrow ^{-} A$ is a corresponding pseudo-derivation.

For $i=k+1$, we divide into cases depending on the rule applied to obtain $s_{k}$. Here we treat the case when it is obtained by (L${\rightarrow }^-$): e.g.

$$\begin{aligned} \frac{A\rightarrow B,\Gamma :\Delta \Rightarrow ^{+}A \qquad B,A\rightarrow B,\Gamma :\Delta \Rightarrow ^{-}C}{A\rightarrow B,\Gamma :\Delta \Rightarrow ^{-}C} {(\text {L}{\rightarrow }^-)} \end{aligned}$$

By the I.H., the pseudo-derivation $d_{k}$ obtained from $d_{a}$ by removing nodes above $s_{k}$ has a corresponding pseudo-derivation $d'_{k}$, whose rightmost initial sequent is $A\rightarrow B,\Gamma :\Delta \Rightarrow ^{+}C$ and is derivable. By Lemma 3 (v), this implies ${\textbf {bGKC}}_{\rightarrow }\vdash B,A\rightarrow B,\Gamma :\Delta \Rightarrow ^{+}C$. So the desired pseudo-derivation corresponding to $d_{k+1}$ is obtained by adding the following inference on top of $d_{k}$:

$$\begin{aligned} \frac{A\rightarrow B,\Gamma :\Delta \Rightarrow ^{+}A \qquad B,A\rightarrow B,\Gamma :\Delta \Rightarrow ^{+}C}{A\rightarrow B,\Gamma :\Delta \Rightarrow ^{+}C} {(\text {L}{\rightarrow }^-)} \end{aligned}$$

where the subderivation up to the left premise may be copied directly from $d_{k+1}$. Other cases are similarly argued, using Lemma 3.

Having constructed $d'_{a}{:}{=}d'_{n}$, it is in general not a derivation because $s'_{n}$ might not be an instance of ($\text {Ax}^-$) or ($\text {Ax}^+$). It is however derivable, so there is a derivation $d'_{b}$ with $s'_{n}$ as the endsequent. Define then $d'$ by attaching $d'_{b}$ on top of $d'_{a}$. Moreover, apply to $d'_{b}$ the same construction we obtained $d'_{a}$ from $d_{a}$. This time, the constructed pseudo-derivation $d_{b}$ is in fact a derivation. To see this, note that its endsequent $d_{n}$ has either the form $p,\Gamma :\Delta \Rightarrow ^{-} p$ or $\Gamma :\Delta ,p\Rightarrow ^{+} p$. Then it suffices to note that none of the rules can affect the *p* or the sign as we go up $d_{b}$ along the rightmost branch. Therefore, defining *d* by attaching $d_{b}$ on top of $d_{a}$ gives the desired pair of *d* and $d'$ which correspond to each other. $\square$

Going back to the example in the introduction, if *A* is a propositional variable then it is possible to construct from each of the derivations the other derivation by the procedure outlined above. In case *A* is not a propositional variable, Theorem 9 still guarantees that corresponding derivations are obtainable.

### Analysis in terms of variables

If two derivations correspond in ${\textbf {bGKC}}_{\rightarrow }$, then their rightmost initial sequents have the form $p,\Gamma :\Delta ,p\Rightarrow ^{-}p$ and $p,\Gamma :\Delta ,p\Rightarrow ^{+}p$; otherwise the sequents fail to be an instance of ($\text {Ax}^-$) or ($\text {Ax}^+$). On the other hand, a derivation of a provable contradiction does not always have an initial sequent of the form. For instance, $(p\rightarrow \mathord {\sim }p)\rightarrow (p\rightarrow \mathord {\sim }p)$ is a provable contradiction but has the following derivation for $\Rightarrow ^{-}$.
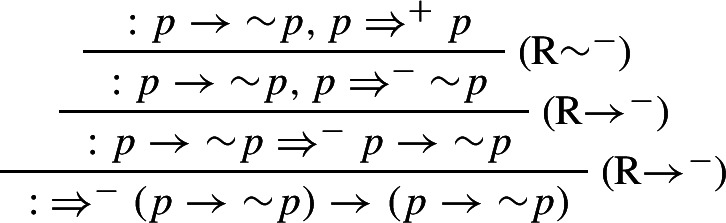


So the correspondence we saw is not a property that all derivations of a provable contradiction automatically enjoy. This then raises a question of whether there is a property all derivations of a provable contradiction satisfy. In order to investigate this, a preliminary notion is introduced. This is an adaptation of the notion of positive/negative/strictly positive formula occurrences [21] to the current setting.

#### Definition 6

We define classes $pos^{+}(A)$, $pos^{-}(A)$, $pos^{+}_{\mathord {\sim }}(A)$, $pos^{-}_{\mathord {\sim }}(A)$, *neg*(*A*), $neg_{\mathord {\sim }}(A)$, *spos*(*A*), $spos_{\mathord {\sim }}(A)$ of occurrences of propositional variables in *A* by the next clauses.
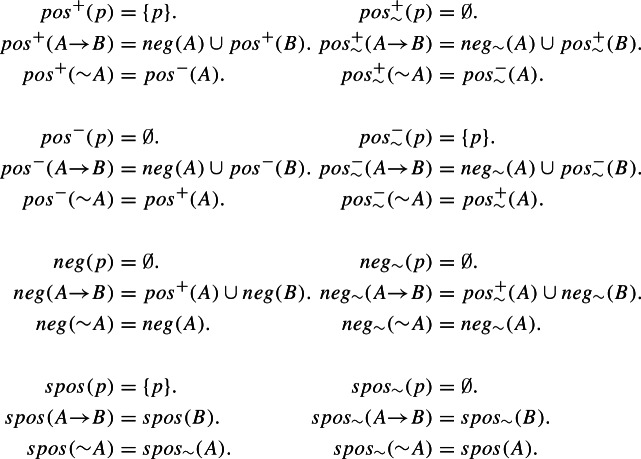
 The classes are naturally extended to multisets of formulas, e.g. $pos^{+}(\Gamma ){:}{=}\{p:p\in pos^{+}(A)\text { for some }A\in \Gamma \}$.

A rough explanation of the classes is as follows: the name of the classes (*pos*/*neg*/*spos*) indicates that it collects propositional variables that occur^8^ (positively/negatively/strictly positively^9^) in a formula; the subscript $\mathord {\sim }$ indicates that it collects propositional variables that occur as e.g. $\mathord {\sim }p$; and the sign of the class corresponds to the distinction of the sign and the two antecedents in a sequent.

The next property will be the focus of this subsection.

#### Definition 7

We shall call a sequent $\Gamma :\Delta \Rightarrow ^{*}C$ *balanced*, if it satisfies the following relationship.

$$\begin{aligned} spos(C)\cup spos_{\mathord {\sim }}(C)\subseteq (pos^{-}_{\mathord {\sim }}(\Gamma )\cup pos^{+}_{\mathord {\sim }}(\Delta )\cup neg_{\mathord {\sim }}(C))\cap (pos^{-}(\Gamma )\cup pos^{+}(\Delta )\cup neg(C)). \end{aligned}$$

Note that the anterior $spos(C)\cup spos_{\mathord {\sim }}(C)$ is always a singleton in the current setting. Then the basic idea of the inclusion above is that the strictly positively occurring variable (say *p*) has to occur either negatively in the succedent or positively in the antecedent, both in the form *p* and $\mathord {\sim }p$ (or something equivalent, in the sense given by (A3)–(A6)). It is also worth emphasising that the sign of a sequent does not play a role in judging balancedness.

The calculus ${\textbf {bGKC}}_{\rightarrow }$ is formulated in such a way as to satisfy the next property.

#### Proposition 10

In ${\textbf {bGKC}}_{\rightarrow }$, the conclusion of a rule is balanced if and only if its (right) premise is.

#### Proof

We shall inspect each of the rules. For an instance of (L$\mathord {\sim }^{-}$):

$$\begin{aligned} \frac{\Gamma :\Delta ,A\Rightarrow ^{*}C}{\mathord {\sim }A,\Gamma :\Delta \Rightarrow ^{*}C} \end{aligned}$$

The premise is balanced if:^10^

$$\begin{aligned} spos(C)\cup spos_{\mathord {\sim }}(C)  &   \subseteq (pos^{-}_{\mathord {\sim }}(\Gamma )\cup pos^{+}_{\mathord {\sim }}(\Delta \cup \{A\})\cup neg_{\mathord {\sim }}(C))\\  &   \cap (pos^{-}(\Gamma )\cup pos^{+}(\Delta \cup \{A\})\cup neg(C)), \end{aligned}$$

whereas the conclusion is balanced if:

$$\begin{aligned} spos(C)\cup spos_{\mathord {\sim }}(C)  &   \subseteq (pos^{-}_{\mathord {\sim }}(\Gamma \cup \{\mathord {\sim }A\})\cup pos^{+}_{\mathord {\sim }}(\Delta )\cup neg_{\mathord {\sim }}(C))\\  &   \cap (pos^{-}(\Gamma \cup \{\mathord {\sim }A\})\cup pos^{+}(\Delta )\cup neg(C)). \end{aligned}$$

The statement then follows since $pos^{-}_{\mathord {\sim }}(\mathord {\sim }A)=pos^{+}_{\mathord {\sim }}(A)$ and $pos^{-}(\mathord {\sim }A)=pos^{+}(A)$. The case for (L$\mathord {\sim }^{+}$) similarly follows since $pos^{+}_{\mathord {\sim }}(\mathord {\sim }A)=pos^{-}_{\mathord {\sim }}(A)$ and $pos^{+}(\mathord {\sim }A)=pos^{-}(A)$.

For an instance of (R$\mathord {\sim }^-$):

$$\begin{aligned} \frac{\Gamma :\Delta \Rightarrow ^{+}C}{\Gamma :\Delta \Rightarrow ^{-}\mathord {\sim }C} \end{aligned}$$

The premise is balanced if:

$$\begin{aligned} spos(C)\cup spos_{\mathord {\sim }}(C)  &   \subseteq (pos^{-}_{\mathord {\sim }}(\Gamma )\cup pos^{+}_{\mathord {\sim }}(\Delta )\cup neg_{\mathord {\sim }}(C))\\  &   \cap (pos^{-}(\Gamma )\cup pos^{+}(\Delta )\cup neg(C)), \end{aligned}$$

whereas the conclusion is balanced if:

$$\begin{aligned} spos(\mathord {\sim }C)\cup spos_{\mathord {\sim }}(\mathord {\sim }C)  &   \subseteq (pos^{-}_{\mathord {\sim }}(\Gamma )\cup pos^{+}_{\mathord {\sim }}(\Delta )\cup neg_{\mathord {\sim }}(\mathord {\sim }C))\\  &   \cap (pos^{-}(\Gamma )\cup pos^{+}(\Delta )\cup neg(\mathord {\sim }C)). \end{aligned}$$

The statement follows as $spos(\mathord {\sim }C)= spos_{\mathord {\sim }}(C)$, $spos_{\mathord {\sim }}(\mathord {\sim }C)=spos(C)$, $neg(\mathord {\sim }C)=neg(C)$ and $neg_{\mathord {\sim }}(\mathord {\sim }C)=neg_{\mathord {\sim }}(C)$. The case for (R$\mathord {\sim }^+$) is analogous.

For an instance of (L${\rightarrow }^{-}$):

$$\begin{aligned} \frac{A\rightarrow B,\Gamma :\Delta \Rightarrow ^{+}A \qquad B,A\rightarrow B,\Gamma :\Delta \Rightarrow ^{*}C}{A\rightarrow B,\Gamma :\Delta \Rightarrow ^{*}C} \end{aligned}$$

The right premise is balanced if:

$$\begin{aligned} spos(C)\cup spos_{\mathord {\sim }}(C)  &   \subseteq (pos^{-}_{\mathord {\sim }}(\Gamma \cup \{A\rightarrow B,B\})\cup pos^{+}_{\mathord {\sim }}(\Delta )\cup neg_{\mathord {\sim }}(C))\\  &   \cap (pos^{-}(\Gamma \cup \{A\rightarrow B,B\})\cup pos^{+}(\Delta )\cup neg(C)), \end{aligned}$$

whereas the conclusion is balanced if:

$$\begin{aligned} spos(C)\cup spos_{\mathord {\sim }}(C)  &   \subseteq (pos^{-}_{\mathord {\sim }}(\Gamma \cup \{A\rightarrow B\})\cup pos^{+}_{\mathord {\sim }}(\Delta )\cup neg_{\mathord {\sim }}(C))\\  &   \cap (pos^{-}(\Gamma \cup \{A\rightarrow B\})\cup pos^{+}(\Delta )\cup neg(C)). \end{aligned}$$

The statement follows as $pos^{-}_{\mathord {\sim }}(B)\subseteq pos^{-}_{\mathord {\sim }}(A\rightarrow B)$ and $pos^{-}(B)\subseteq pos^{-}(A\rightarrow B)$. The case for (L${\rightarrow }^+$) follows analogously.

For an instance of (R${\rightarrow }^-$):

$$\begin{aligned} \frac{\Gamma :\Delta ,A\Rightarrow ^{-}C}{\Gamma :\Delta \Rightarrow ^{-}A\rightarrow C} \end{aligned}$$

The right premise is balanced if:

$$\begin{aligned} spos(C)\cup spos_{\mathord {\sim }}(C)  &   \subseteq (pos^{-}_{\mathord {\sim }}(\Gamma )\cup pos^{+}_{\mathord {\sim }}(\Delta \cup \{A\})\cup neg_{\mathord {\sim }}(C))\\  &   \cap (pos^{-}(\Gamma )\cup pos^{+}(\Delta \cup \{A\})\cup neg(C)), \end{aligned}$$

whereas the conclusion is balanced if:

$$\begin{aligned} spos(A\rightarrow C)\cup spos_{\mathord {\sim }}(A\rightarrow C)  &   \subseteq (pos^{-}_{\mathord {\sim }}(\Gamma )\cup pos^{+}_{\mathord {\sim }}(\Delta )\cup neg_{\mathord {\sim }}(A\rightarrow C))\\  &   \cap (pos^{-}(\Gamma )\cup pos^{+}(\Delta )\cup neg(A\rightarrow C)). \end{aligned}$$

The statement follows as $neg_{\mathord {\sim }}(A\rightarrow C)=pos^{+}_{\mathord {\sim }}(A)\cup neg_{\mathord {\sim }}(C)$ and $neg(A\rightarrow C)=pos^{+}(A)\cup neg(C)$. The case for (R${\rightarrow }^+$) is analogous. $\square$

Hence if the endsequent of a derivation is balanced, then we can infer that the rightmost initial sequent must be balanced as well. The question then is when we have such an endsequent. We will relate this question to provable contradiction, for which we need a couple of semantical lemmas.

#### Definition 8

Let $\mathcal {F}=(\{w\},\{(w,w)\})$ and *p* be a propositional variable. We define two types of models $\mathcal {M}^{p}_{1}=(\mathcal {F},\mathcal {V}^{+}_{1},\mathcal {V}^{-}_{1})$ and $\mathcal {M}^{p}_{2}=(\mathcal {F},\mathcal {V}^{+}_{2},\mathcal {V}^{-}_{2})$ such that $\mathcal {V}^{+}_{1}(q)=\mathcal {V}^{-}_{2}(q)=\emptyset$ if $p\equiv q$, and $=\{w\}$ otherwise; $\mathcal {V}^{-}_{1}(q)=\mathcal {V}^{+}_{2}(q)=\{w\}$ for all *q*.

#### Lemma 11

The following statements hold.

(i) If $$p\notin pos^{+}(A)$$, then $$\mathcal {M}^{p}_{1},w\Vdash ^{+}A$$.
(ii) If $p\notin pos^{+}_{\mathord {\sim }}(A)$, then $\mathcal {M}^{p}_{2},w\Vdash ^{+}A$.

#### Proof

We show by induction on the complexity of *A*. We shall check the cases for (i), and the cases for (ii) are analogous.

If $A\equiv q$, then since $pos^{+}(q)=\{q\}$, $p\notin pos^{+}(q)$ implies $p\not \equiv q$. Thus $\mathcal {M}^{p}_{1},w\Vdash ^{+}q$.

If $A\equiv B\rightarrow C$ then $p\notin pos^{+}(B\rightarrow C)=neg(B)\cup pos^{+}(C)$ in particular implies $p\notin pos^{+}(C)$. Hence by the I.H. it follows that $\mathcal {M}^{p}_{1},w\Vdash ^{+} C$. So $\mathcal {M}^{p}_{1},w\Vdash ^{+}B\rightarrow C$.

If $A\equiv \mathord {\sim }q$, then $\mathcal {M},w\Vdash ^{+}\mathord {\sim }q$ as $w\in \mathcal {V}^{-}(q)$ for all *q*.

If $A\equiv \mathord {\sim }(B\rightarrow C)$, then $p\notin pos^{+}(\mathord {\sim }(B\rightarrow C))=pos^{-}(B\rightarrow C)=neg(B)\cup pos^{-}(C)=neg(B)\cup pos^{+}(\mathord {\sim }C)$ implies $p\notin pos^{+}(\mathord {\sim }C)$. By the I.H. this implies $\mathcal {M}^{p}_{1},w\Vdash ^{+}\mathord {\sim }C$ and so $\mathcal {M}^{p}_{1},w\Vdash ^{+}\mathord {\sim }(B\rightarrow C)$.

If $A\equiv \mathord {\sim }\mathord {\sim }B$, then $p\notin pos^{+}(\mathord {\sim }\mathord {\sim }B)=pos^{-}(\mathord {\sim }B)= pos^{+}(B)$ implies by the I.H. $\mathcal {M},w\Vdash ^{+}B$ and hence $\mathcal {M},w\Vdash ^{+}\mathord {\sim }\mathord {\sim }B$. $\square$

#### Lemma 12

Assume $p\in spos(A)\cup spos_{\mathord {\sim }}(A)$. Then the next statements hold:

(i) If $p\notin neg(A)$ then $\mathcal {M}^{p}_{1},w\nVdash ^{+}A$ or $\mathcal {M}^{p}_{1},w\nVdash ^{-}A$.
(ii) If $p\notin neg_{\mathord {\sim }}(A)$ then $\mathcal {M}^{p}_{2},w\nVdash ^{+}A$ or $\mathcal {M}^{p}_{2},w\nVdash ^{-}A$.

#### Proof

By induction on the complexity of *A*. The arguments for (i) and (ii) are similar, and here we concentrate on the former case.

If $A\equiv q$, then $p\in spos(A)\cup spos_{\mathord {\sim }}(A)$ implies $p\equiv q$ and so $\mathcal {M}^{p}_{1},w\nVdash ^{+}q$.

If $A\equiv B\rightarrow C$, then the assumption implies that $p\in spos(C)\cup spos_{\mathord {\sim }}(C)$. So by the I.H. $p\notin neg(C)$ implies that $\mathcal {M}^{p}_{1},w\nVdash ^{+}C$ or $\mathcal {M}^{p}_{1},w\nVdash ^{-}C$. Also, $p\notin neg(B\rightarrow C)= pos^{+}(B)\cup neg(C)$ implies that $p\notin pos^{+}(B)$ and $p\notin neg(C)$. By Lemma 11, the former conjunct implies $\mathcal {M}^{p}_{1}\Vdash ^{+}B$. On the other hand, the latter conjunct implies $\mathcal {M}^{p}_{1},w\nVdash ^{+}C$ or $\mathcal {M}^{p}_{1},w\nVdash ^{-}C$ from the above observation. Thus either $\mathcal {M}^{p}_{1},w\nVdash ^{+}B\rightarrow C$ or $\mathcal {M}^{p}_{1},w\nVdash ^{-}B\rightarrow C$.

If $A\equiv \mathord {\sim }B$, then $p\in spos(\mathord {\sim }B)\cup spos_{\mathord {\sim }}(\mathord {\sim }B)$ implies $p\in spos(B)\cup spos_{\mathord {\sim }}(B)$. So by the I.H. $p\notin neg(B)$ implies $\mathcal {M}^{p}_{1},w\nVdash ^{+}B$ or $\mathcal {M}^{p}_{1},w\nVdash ^{-}B$. Now, $p\notin neg(\mathord {\sim }B)$ implies $p\notin neg(B)$. Therefore either $\mathcal {M}^{p}_{1},w\nVdash ^{-}\mathord {\sim }B$ or $\mathcal {M}^{p}_{1},w\nVdash ^{+}\mathord {\sim }B$. $\square$

We can now conclude that sequents for a contradictory formula $A$ are balanced, which in such a case means $spos(A)\cup spos_{\mathord {\sim }}(A)\subseteq neg(A)\cap neg_{\mathord {\sim }}(A)$.

#### Theorem 13

If ${\textbf {bGKC}}_{\rightarrow }\vdash \ :\ \Rightarrow ^{+}A$ and ${\textbf {bGKC}}_{\rightarrow }\vdash \ :\ \Rightarrow ^{-}A$, then the sequents are balanced.

#### Proof

We show the contrapositive of the statement. Suppose $\ :\ \Rightarrow ^{+}A$ (or $\Rightarrow ^{-}$) is not balanced, i.e. $spos(A)\cup spos_{\mathord {\sim }}(A)\nsubseteq neg(A)\cap neg_{\mathord {\sim }}(A)$ in this case. Then there is $p\in spos(A)\cup spos_{\mathord {\sim }}(A)$ such that $p\notin neg(A)\cap neg_{\mathord {\sim }}(A)$. Then either $p\notin neg(A)$ or $p\notin neg_{\mathord {\sim }}(A)$, and in each case $\nvDash A$ or $\nvDash \mathord {\sim }A$ by Lemma 12. Hence either ${\textbf {bGKC}}_{\rightarrow }\nvdash \ :\ \Rightarrow ^{+}A$ or ${\textbf {bGKC}}_{\rightarrow }\nvdash \ :\ \Rightarrow ^{-}A$ from Theorem 8. $\square$

Combining Proposition 10 and Theorem 13, we obtain a method to reason consistently, by starting a derivation with a specific type of initial sequent.

#### Corollary 14

The following statements hold.

(i) If a derivation of ${\textbf {bGKC}}_{\rightarrow }$ begins with an instance $p, \Gamma :\Delta \Rightarrow ^{-}p$ of (${Ax}^-$) such that $p\notin pos^{-}(\Gamma )\cup pos^{+}(\Delta )$ as the rightmost initial sequent, then it never derives a provable contradiction.
(ii) If a derivation of ${\textbf {bGKC}}_{\rightarrow }$ begins with an instance $\Gamma :\Delta ,p\Rightarrow ^{+}p$ of (${Ax}^+$) such that $p\notin pos^{-}_{\mathord {\sim }}(\Gamma )\cup pos^{+}_{\mathord {\sim }}(\Delta )$ as the rightmost initial sequent, then it never derives a provable contradiction.

#### Proof

In each case, the rightmost initial sequent is not balanced, so by Proposition 10 the endsequent must be unbalanced as well. On the other hand, a provable contradiction must be balanced, by Theorem 13. $\square$

## The full propositional case

### Provable contradictions in **bGKC**

The first objective of this section is to generalise Theorem 9 for **bGKC**. There is a problem in applying the argument of the last section, because there are rules such as (R$\vee ^{+}$) which are not invertible. The property of invertibility is used crucially in the implication-negation case. It is for this reason necessary to restrict our attention to a certain class of provable contradictions when we stick to **bGKC**.

This requires us to talk of an occurrence of subformulas in certain positions of a formula. We shall follow Troelstra and van Dalen [20] and expand $\mathcal {L}$ with a special type of atomic formula called *schema* $\$$ indicating a specific position in a formula.

#### Definition 9

We define the next types of *formula contexts*.

$$\begin{aligned} \mathcal {P}^{+}&{::}{=}&\$\ |\ A\circ \mathcal {P}^{+}\ |\ \mathcal {P}^{+}\circ A\ |\ A\rightarrow \mathcal {P}^{+}\ |\ \mathcal {N}\rightarrow A\ |\ \mathord {\sim }\mathcal {P}^{-}.\\ \mathcal {P}^{-}&{::}{=}&A\circ \mathcal {P}^{-}\ |\ \mathcal {P}^{-}\circ A\ |\ A\rightarrow \mathcal {P}^{-}\ |\ \mathcal {N}\rightarrow A\ |\ \mathord {\sim }\mathcal {P}^{+}.\\ \mathcal {P}^{+}_{\mathord {\sim }}&{::}{=}&A\circ \mathcal {P}^{+}_{\mathord {\sim }}\ |\ \mathcal {P}^{+}_{\mathord {\sim }}\circ A\ |\ A\rightarrow \mathcal {P}^{+}_{\mathord {\sim }}\ |\ \mathcal {N}_{\mathord {\sim }}\rightarrow A\ |\ \mathord {\sim }\mathcal {P}^{-}_{\mathord {\sim }}.\\ \mathcal {P}^{-}_{\mathord {\sim }}&{::}{=}&\$\ |\ A\circ \mathcal {P}^{-}_{\mathord {\sim }}\ |\ \mathcal {P}^{-}_{\mathord {\sim }}\circ A\ |\ A\rightarrow \mathcal {P}^{-}_{\mathord {\sim }}\ |\ \mathcal {N}_{\mathord {\sim }}\rightarrow A\ |\ \mathord {\sim }\mathcal {P}^{+}_{\mathord {\sim }}.\\ \mathcal {N}&{::}{=}&A\circ \mathcal {N}\ |\ \mathcal {N}\circ A\ |\ A\rightarrow \mathcal {N}\ |\ \mathcal {P}^{+}\rightarrow A\ |\ \mathord {\sim }\mathcal {N}.\\ \mathcal {N}_{\mathord {\sim }}&{::}{=}&A\circ \mathcal {N}_{\mathord {\sim }}\ |\ \mathcal {N}_{\mathord {\sim }}\circ A\ |\ A\rightarrow \mathcal {N}_{\mathord {\sim }}\ |\ \mathcal {P}^{+}_{\mathord {\sim }}\rightarrow A\ |\ \mathord {\sim }\mathcal {N}_{\mathord {\sim }}.\\ \mathcal{S}\mathcal{P}&{::}{=}&\$\ |\ A\circ \mathcal{S}\mathcal{P}\ |\ \mathcal{S}\mathcal{P}\circ A\ |\ A\rightarrow \mathcal{S}\mathcal{P}\ |\ \mathord {\sim }\mathcal{S}\mathcal{P}_{\mathord {\sim }}.\\ \mathcal{S}\mathcal{P}_{\mathord {\sim }}&{::}{=}&A\circ \mathcal{S}\mathcal{P}_{\mathord {\sim }}\ |\ \mathcal{S}\mathcal{P}_{\mathord {\sim }}\circ A\ |\ A\rightarrow \mathcal{S}\mathcal{P}_{\mathord {\sim }}\ |\ \mathord {\sim }\mathcal{S}\mathcal{P}. \end{aligned}$$

where $$\circ \in \{\wedge ,\vee \}$$.

We say a formula *B* *occurs in a formula* *A* *with respect to a class* $\mathcal {C}$ if there is an expanded formula $C[\$]$ such that the result of substituting $\$$ with *B* is *A*: i.e. $A\equiv C[B]$. The sets defined in Definition 6 are specific cases of the notion: e.g. $pos^{+}(A)$ is the set of all propositional variables occurring w.r.t. $\mathcal {P}^{+}$ (in $\mathcal {L}_{\rightarrow }$).

In order to replicate the method of constructing corresponding branches in the current setting, we shall appeal to the disjunction and constructible falsity property of Rasiowa-Harrop type (cf. e.g. [21]), and in particular we must assure that these properties are available whenever necessary. To guarantee this, we state a few lemmas.

#### Lemma 15

$$\mathcal{S}\mathcal{P}\subseteq \mathcal {P}^{+}\cap \mathcal {P}^{-}_{\mathord {\sim }}$$ and $$\mathcal{S}\mathcal{P}_{\mathord {\sim }}\subseteq \mathcal {P}^{-}\cap \mathcal {P}^{+}_{\mathord {\sim }}$$.

#### Proof

By induction on the complexity of formulas, with both cases treated simultaneously. For instance, if $A\in \mathcal{S}\mathcal{P}$ has the form $\mathord {\sim }B$ for $B\in \mathcal{S}\mathcal{P}_{\mathord {\sim }}$, then by I.H, $B\in \mathcal {P}^{-}\cap \mathcal {P}^{+}_{\mathord {\sim }}$ and so $A\in \mathcal {P}^{+}\cap \mathcal {P}^{-}_{\mathord {\sim }}$.

$$\square $$

#### Lemma 16

If $$\textbf{bGKC}\vdash \Gamma :\Delta \Rightarrow ^{*}A$$ and

1. conjunctions are not occurring in $${\left\{ \begin{array}{ll} \Gamma \text { w.r.t. }\mathcal{S}\mathcal{P}\\ \Delta \text { w.r.t. }\mathcal{S}\mathcal{P}_{\mathord {\sim }}\\ A \text { w.r.t. }\mathcal {N}_{\mathord {\sim }} \end{array}\right. }$$
2. disjunctions are not occurring in $${\left\{ \begin{array}{ll} \Gamma \text { w.r.t. }\mathcal{S}\mathcal{P}_{\mathord {\sim }}\\ \Delta \text { w.r.t. }\mathcal{S}\mathcal{P}\\ A \text { w.r.t. }\mathcal {N} \end{array}\right. }$$

then the same property is satisfied by all sequents in a derivation, except for the left premises of (L${\rightarrow }^{-}$) and (L${\rightarrow }^{+}$) and the sequents above them.

#### Proof

By induction on the depth of derivation. If the derivation is an instance of ($\text {Ax}^+$) or ($\text {Ax}^-$), then there is nothing to show.

Suppose that the derivation ends with an instance of (L${\rightarrow }^-$).

$$\begin{aligned} \frac{B\rightarrow C,\Gamma :\Delta \Rightarrow ^{+}B \qquad C,B\rightarrow C,\Gamma :\Delta \Rightarrow ^{*}D}{B\rightarrow C,\Gamma :\Delta \Rightarrow ^{*}D} {(\text {L}{\rightarrow }^-)} \end{aligned}$$

Then it suffices to show that conjunctions do not occur w.r.t. $\mathcal{S}\mathcal{P}$ in *C*, because then the right premise satisfies the desired properties, and by the I.H. the same applies to the sequents above it. So suppose a conjunction $E\wedge F$ occurs in *C* w.r.t. $\mathcal{S}\mathcal{P}$, i.e. there is $C'[\$]\in \mathcal{S}\mathcal{P}$ such that $C'[E\wedge F]\equiv C$. But then $(B\rightarrow C')[\$]\in \mathcal{S}\mathcal{P}$, and as a result conjunction occurs in $B\rightarrow C$ w.r.t. $\mathcal{S}\mathcal{P}$, a contradiction. Hence conjunctions do not occur w.r.t. $\mathcal{S}\mathcal{P}$ in *C*. Similarly, if a disjunction occurs in *C* w.r.t $\mathcal{S}\mathcal{P}_{\mathord {\sim }}$ then so does it in $B\rightarrow C$ w.r.t $\mathcal{S}\mathcal{P}_{\mathord {\sim }}$, but this is not possible.

Suppose that the derivation in an instance of (R${\rightarrow }^-$).

$$\begin{aligned} \frac{\Gamma :\Delta ,B\Rightarrow ^{-}C}{\Gamma :\Delta \Rightarrow ^{-}B\rightarrow C} {(\text {R}{\rightarrow }^-)} \end{aligned}$$

If a conjunction occurs in *B* w.r.t. $\mathcal{S}\mathcal{P}_{\mathord {\sim }}$, then it occurs in *B* w.r.t. $\mathcal {P}^{+}_{\mathord {\sim }}$ as well, by Lemma 15. Consequently it must occur in $B\rightarrow C$ w.r.t. $\mathcal {N}_{\mathord {\sim }}$ contrary to the I.H. Similarly, if a disjunction occurs in *B* w.r.t. $\mathcal{S}\mathcal{P}$, then it must occur in $B\rightarrow C$ w.r.t. $\mathcal {N}$. Furthermore, if a conjunction (disjunction) occurs in *C* w.r.t. $\mathcal {N_{\mathord {\sim }}}$ ($\mathcal {N}$) then so does it in $B\rightarrow C$ w.r.t. the same class, a contradiction. The cases for (L$\rightarrow ^{+}$) and (R$\rightarrow ^{+}$) are analogous.

If the derivation ends with an instance of (L$\mathord {\sim }^{-}$):

$$\begin{aligned} \frac{\Gamma :\Delta ,B\Rightarrow ^{*}C}{\mathord {\sim }B,\Gamma :\Delta \Rightarrow ^{*}C} {(\text {L}\mathord {\sim }^{-})} \end{aligned}$$

then a disjunction (conjunction) occurring in *B* w.r.t. $\mathcal{S}\mathcal{P}_{\mathord {\sim }}$ ($\mathcal{S}\mathcal{P}$) implies its occurring in $\mathord {\sim }B$ w.r.t. $\mathcal{S}\mathcal{P}$ ($\mathcal{S}\mathcal{P}_{\mathord {\sim }}$) contrary to the I.H..

Suppose a derivation ends with an instance of (R$\mathord {\sim }^{-}$):

$$\begin{aligned} \frac{\Gamma :\Delta \Rightarrow ^{+}C}{\Gamma :\Delta \Rightarrow ^{-}\mathord {\sim }C} {(R\mathord {\sim }^{-})} \end{aligned}$$

If a conjunction (disjunction) occurs in *C* w.r.t $\mathcal {N}_{\mathord {\sim }}$ ($\mathcal {N}$), then it occurs in $\mathord {\sim }C$ w.r.t $\mathcal {N}_{\mathord {\sim }}$ ($\mathcal {N}$), contradicting the I.H. The cases for (L$\mathord {\sim }^{+}$) and (R$\mathord {\sim }^{+}$) are analogous.

The derivation cannot end with (L$\wedge ^{-}$) or (R$\vee ^{+}$) because then a conjunction (disjunction) occurs in the negative (positive) antecedent of the endsequent w.r.t. $\mathcal{S}\mathcal{P}$. The remaining cases are argued similarly to the above cases. $\square$

As shown in [22], the system **C** satisfies the *constructible falsity /disjunction property*, namely that a falsified conjunction/verified disjunction always has one of the conjuncts/disjuncts as the witness. The same properties hold with a certain class of assumptions; in the current setting this is expressed as follows.

#### Lemma 17

The following statements hold.

(i) If $$\textbf{bGKC}\vdash \Gamma :\Delta \Rightarrow ^{-}A\wedge B$$ and
  1. conjunctions are not occurring in $${\left\{ \begin{array}{ll} \Gamma \text { w.r.t. }\mathcal{S}\mathcal{P}\\ \Delta \text { w.r.t. }\mathcal{S}\mathcal{P}_{\mathord {\sim }}\\ \end{array}\right. }$$
  2. disjunctions are not occurring in $${\left\{ \begin{array}{ll} \Gamma \text { w.r.t. }\mathcal{S}\mathcal{P}_{\mathord {\sim }}\\ \Delta \text { w.r.t. }\mathcal{S}\mathcal{P}\\ \end{array}\right. }$$
  then $$\textbf{bGKC}\vdash \Gamma :\Delta \Rightarrow ^{-}A$$ or $$\textbf{bGKC}\vdash \Gamma :\Delta \Rightarrow ^{-}B$$.
(ii) If $\textbf{bGKC}\vdash \Gamma :\Delta \Rightarrow ^{+}A\vee B$ and 1., 2. are satisfied, then $\textbf{bGKC}\vdash \Gamma :\Delta \Rightarrow ^{+}A$ or $\textbf{bGKC}\vdash \Gamma :\Delta \Rightarrow ^{+}B$.

#### Proof

(i) If we go up a derivation of $\textbf{bGKC}\vdash \Gamma :\Delta \Rightarrow ^{-}A\wedge B$, then at each step exactly one of the premises keeps the succedent (including the sign), as well as preserving 1. and 2., except for (R$\wedge ^{-}$): we do not meet (L$\wedge ^{-}$) or (L$\vee ^+$) because of 1. and 2. Following such nodes, we eventually have to reach an instance of (R$\wedge ^{-}$) since a conjunction cannot occur in the succedent in an initial sequent. The succedent of the premise is either *A* or *B* with the sign +; we can rewrite the derivation by skipping the application of (R$\wedge ^{-}$). The subsequent applications of rules remain correct, and the new endsequent has the desired form. (ii) is similarly argued. $\square$

The correspondence of derivations in **bGKC** is then seen to hold with respect to the next class of formulas.

#### Theorem 18

Assume $\textbf{bGKC}\vdash \ :\ \Rightarrow ^{+}A$ and $\textbf{bGKC}\vdash \ :\ \Rightarrow ^{-}A$, where conjunctions do not occur in *A* w.r.t. $\mathcal {N}_{\mathord {\sim }}$ and disjunctions do not occur w.r.t. $\mathcal {N}$. Then there are derivations of the sequents which correspond.

#### Proof

Take a derivation of $d_{a}$ of $\ :\ \Rightarrow ^{+}A$ (one may also take start with a derivation of $\ :\ \Rightarrow ^{-}A$). As in the implication-negation case, we shall first create a pseudo-derivation $d'_{a}$ which corresponds to $d_{a}$ except that there is a branch whose initial sequent, while derivable, may not be an instance of ($\text {Ax}^-$) or ($\text {Ax}^+$).

We shall define a branch $s_{0},\ldots ,s_{n}$ in $d_{a}$, and in each step *i* we simultaneously define a pseudo-derivation $d'_{i}$ of $\ :\ \Rightarrow ^{-}A$ with a branch $s'_{0},\ldots ,s'_{i}$ corresponding to $s_{0},\dots ,s_{i}$ in the pseudo-derivation $d_{i}$ obtained from $d_{a}$ by removing nodes above $s_{i}$.

When $i=0$, take $s_{0}$ to be $\ :\ \Rightarrow ^{+}A$ and the branch $d'_{0}$ is defined with a single node $s'_{0}$ of a sequent $\ :\ \Rightarrow ^{-}A$.

For the inductive step, assume that $s_{i}$ and $s'_{i}$ are defined and that there is a pseudo-derivation $d'_{i}$ with a branch $s'_{0},\ldots ,s'_{i}$ corresponding to a branch $s_{0},\ldots ,s_{i}$ in a pseudo-derivation obtained from $d_{a}$ by removing nodes above $s_{i}$. We need to then choose $s_{i+1}$ and define a pseudo-derivation $d'_{i+1}$ of $\ :\ \Rightarrow ^{-}A$ with a branch $s'_{0},\ldots ,s'_{i+1}$ corresponding to a branch $s_{0},\ldots ,s_{i+1}$ in a pseudo-derivation obtained from $d_{a}$ by removing nodes above $s_{i+1}$. We divide into cases depending on the rule applied to obtain $s_{i}$. Given Theorem 9, we can concentrate on the cases for the rules of conjunction and disjunction: for the cases of (L${\rightarrow }^{-}$) and (L${\rightarrow }^{+}$), the right premise can be chosen as $s_{i+1}$, and as a consequence the conditions to apply Lemma 17 are satisfied for both $s_{i}$ and $s'_{i}$ because of our assumption on *A* and Lemma 16. This also implies that $s_{i}$ is not obtained by (L$\wedge ^{-}$) or (L$\vee ^{+}$).

If $s_{i}$ is obtained by (R$\wedge ^{-}$), e.g.:

$$\begin{aligned} \frac{\Gamma :\Delta \Rightarrow ^{-}C_{1}}{\Gamma :\Delta \Rightarrow ^{-}C_{1}\wedge C_{2}} {(\text {R}\wedge ^{-})} \end{aligned}$$

then choose the premise to be $s_{i+1}$. By the I.H., $s'_{i}$ is $\Gamma :\Delta \Rightarrow ^{+}C_{1}\wedge C_{2}$, and by Lemma 3 (xi) $\textbf{bGKC}\vdash \Gamma :\Delta \Rightarrow ^{+}C_{1}$ and $\textbf{bGKC}\vdash \Gamma :\Delta \Rightarrow ^{+}C_{2}$. Take the former sequent as $s'_{i+1}$ and the pseudo-derivation is obtained by attaching to the pseudo-derivation up to $s'_{i}$ with a new step:

$$\begin{aligned} \frac{\Gamma :\Delta \Rightarrow ^{+}C_{1} \qquad \Gamma :\Delta \Rightarrow ^{+}C_{2}}{\Gamma :\Delta \Rightarrow ^{+}C_{1}\wedge C_{2}} {(\text {R}\wedge ^{+})} \end{aligned}$$

where the subderivation above the right premise is arbitrary taken.

If $s_{i}$ is obtained by (L$\wedge ^{+}$), e.g.:

$$\begin{aligned} \frac{\Gamma :\Delta ,B,C\Rightarrow ^{+}D}{\Gamma :\Delta ,B\wedge C\Rightarrow ^{+}D} {(\text {L}\wedge ^{+})} \end{aligned}$$

then choose the premise to be $s_{i+1}$. By the I.H. $s'_{i}$ is $\Gamma :\Delta ,B\wedge C\Rightarrow ^{-}D$, and by Lemma 3 (x) $\textbf{bGKC}\vdash \Gamma :\Delta ,B, C\Rightarrow ^{-}D$. Choose the sequent as $s'_{i+1}$ and $d'_{i+1}$ is obtained by adding to $d_{i+1}$:

$$\begin{aligned} \frac{\Gamma :\Delta ,B,C\Rightarrow ^{-}D}{\Gamma :\Delta ,B\wedge C\Rightarrow ^{-}D} {(\text {L}\wedge ^{+})} \end{aligned}$$

If $s_{i}$ is obtained by (R$\wedge ^{+}$), e.g.:

$$\begin{aligned} \frac{\Gamma :\Delta \Rightarrow ^{+}B \qquad \Gamma :\Delta \Rightarrow ^{+}C}{\Gamma :\Delta \Rightarrow ^{+}B\wedge C} {(\text {R}\wedge ^{+})} \end{aligned}$$

then by the I.H. $s'_{i}$ is $\Gamma :\Delta \Rightarrow ^{-}B\wedge C$, and by Lemma 17 either $\textbf{bGKC}\vdash \Gamma :\Delta \Rightarrow ^{-}B$ or $\textbf{bGKC}\vdash \Gamma :\Delta \Rightarrow ^{-}C$. Suppose, without loss of generality, the former. Then we choose $s_{i}$ to be $\Gamma :\Delta \Rightarrow ^{+}B$ and $s'_{i}$ to be $\Gamma :\Delta \Rightarrow ^{-}B$. The new pseudo-derivation is obtained by attaching:

$$\begin{aligned} \frac{\Gamma :\Delta \Rightarrow ^{-}B}{\Gamma :\Delta \Rightarrow ^{-}B\wedge C} {(\text {R}\wedge ^{-})} \end{aligned}$$

The cases for disjunction are analogous to one of the above cases.

Once a pseudo-derivation $d'_{a}{:}{=}d'_{n}$ corresponding to $d_{a}$ is defined, we apply the same procedure as Theorem 9 and take a derivation $d'_{b}$ of $s'_{n}$ and then apply the above procedure to obtain a pseudo-derivation $d_{b}$ corresponding to it. Since our construction of the corresponding branch in $d_{b}$ avoids left premises of (L$\rightarrow ^{*}$), the sequents in the branch remain an instance of ($\text {Ax}^{-}$) or ($\text {Ax}^{+}$), so $d_{b}$ is in fact a derivation. Hence the desired derivations *d* and $d'$ are obtained by attaching $d_{b}$ ($d'_{b}$) to $d_{a}$ ($d'_{a}$), respectively.

$$\square $$

#### Example 1

A simple example of provable contradictions falling under the scope of Theorem 18 is $\textbf{bGKC}\vdash \ :\ \Rightarrow ^{*}(p\wedge \mathord {\sim }p)\rightarrow (p\vee \mathord {\sim }p)$ for $*\in \{1,2\}$. The derivations below for instance exhibits a correspondence, and one can be contained from the other by the procedure outlined in the above proof.
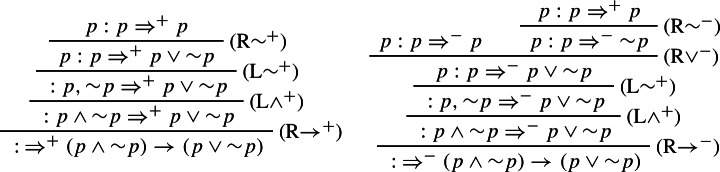


### Using tableaux

In order to obtain Theorem 18, we had to appeal to the constructible falsity and disjunction property which complemented the lack of invertibility for some rules. This resulted in restricting our attention to a class of formulas which allowed us to use the properties when constructing pseudo-derivations.

In this subsection, we shall observe that it is possible to remove this restriction with a simple expansion of our framework. The framework we shall use was introduced by G. G. Mints [13]. In this framework, the basis unit of derivation is not a sequent but a *tableau* which is a finite non-empty list of sequents (i.e. a kind of hypersequent). A tableau may be depicted as $\sigma |\Gamma :\Delta \Rightarrow ^{*}A|\tau$, where $\sigma ,\tau$ are finite (possibly empty) lists of sequents. The tableau calculus we shall consider is defined by modifying **bGKC**.

#### Definition 10

A tableau calculus **bTKC** is defined from **bGKC** by changing sequents in a rule into tableaux, except for (L$\rightarrow ^{*}$), (R$\wedge ^*$) and (R$\vee ^*$). We shall use ($\text {tAx}^{-}$), (tR$\mathord {\sim }^{+}$) etc. for the names of the tableau rules: e,g, (L$\vee ^{+}$) is the next rule:

$$\begin{aligned} \frac{\sigma |\Gamma :\Delta ,A\vee B,A\Rightarrow ^{*} C|\tau \qquad \sigma |\Gamma :\Delta ,A\vee B,B\Rightarrow ^{*} C|\tau }{\sigma |\Gamma :\Delta ,A\vee B\Rightarrow ^{*}C|\tau } {(tL{\vee }^+)} \end{aligned}$$

For (L$\rightarrow ^{*}$), (R$\wedge ^*$) and (R$\vee ^*$), we have the next modifications.
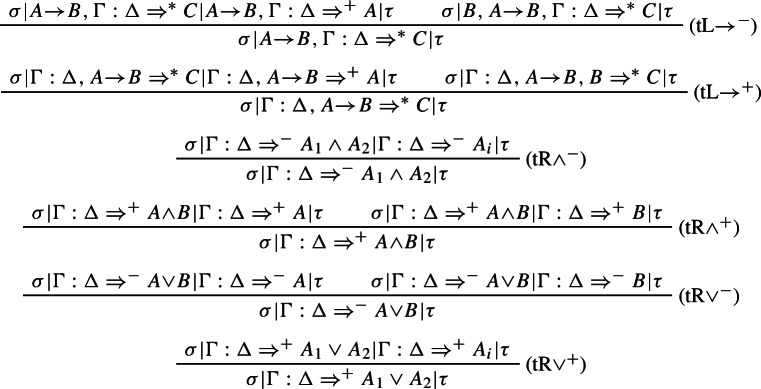


A derivation in **bTKC** is defined analogously to a derivation in **bGKC**. We next present basic properties of **bTKC** that are standard for tableau calculi.

#### Lemma 19

The following rules are depth-preserving admissible in **bTKC**:

$$\begin{aligned}  &   \frac{\sigma |\Gamma :\Delta \Rightarrow ^{*}C|\tau }{\sigma |A,\Gamma :\Delta \Rightarrow ^{*}C|\tau } {(tLW^{-})} \frac{\sigma |\Gamma :\Delta \Rightarrow ^{*}C|\tau }{\sigma |\Gamma :\Delta ,A\Rightarrow ^{*}C|\tau } {(tLW^{+})}\\  &   \frac{\sigma |\Gamma :\Delta \Rightarrow ^{*}A|\tau }{ \sigma |\sigma '|\Gamma :\Delta \Rightarrow ^{*}A|\tau '|\tau } {(tW)}. \end{aligned}$$

#### Proof

By induction on the depth of derivations. For ($\text {tLW}^-$) and ($\text {tLW}^+$), we have to apply the I.H. twice when the derivation ends with a rule which reduces the number of sequents in a tableaux, for example (tR$\wedge ^-$). The applicability of the I.H. for the second time is justified by the depth-preservation.

$$\square $$

#### Proposition 20

$\textbf{bTKC}\vdash \Gamma :\Delta \Rightarrow ^{*}A$ if and only if $\textbf{bGKC}\vdash \Gamma :\Delta \Rightarrow ^{*}A$.

#### Proof

For the ‘only if’ direction, we shall show by induction on the depth of derivations in **bTKC** that the derivability of a tableau in **bTKC** implies the derivability of a sequent from the tableau in **bGKC**. For ($\text {tAx}^*$), we can take the displayed sequent.

If the last rule applied is an instance of (tL${\rightarrow }^{-}$):

$$\begin{aligned} \frac{\sigma |A{\rightarrow }B,\Gamma :\Delta \Rightarrow ^{*}C|A{\rightarrow }B,\Gamma :\Delta \Rightarrow ^{+} A|\tau \qquad \sigma |B,A{\rightarrow }B,\Gamma :\Delta \Rightarrow ^{*}C|\tau }{\sigma |A{\rightarrow }B,\Gamma :\Delta \Rightarrow ^{*}C|\tau } {(\text {tL}{\rightarrow }^-)} \end{aligned}$$

Then by the I.H., either (i) a sequent in $\sigma$ or $\tau$ is derivable (in **bGKC**), (ii) $\Gamma :\Delta \Rightarrow ^{*}C$ is derivable, or (iii) both $A{\rightarrow }B,\Gamma :\Delta \Rightarrow ^{+} A$ and $B,A{\rightarrow }B,\Gamma :\Delta \Rightarrow ^{*}C$ are derivable. In the cases (i) and (ii), the same sequent can be taken as a derivable sequent for the conclusion. In the case (iii), we can apply (L${\rightarrow }^{-}$) to obtain a derivable sequent $A{\rightarrow }B,\Gamma :\Delta \Rightarrow ^{*}C$ for the conclusion. The argument is similar for other cases.

For the ‘if’ case, we use induction on the depth of derivations in **bGKC**. For instance, if the last rule is an instance of (L${\rightarrow }^{-}$):

$$\begin{aligned} \frac{A{\rightarrow }B,\Gamma :\Delta \Rightarrow ^{+} A \qquad B,A{\rightarrow }B,\Gamma :\Delta \Rightarrow ^{*}C}{A{\rightarrow }B,\Gamma :\Delta \Rightarrow ^{*}C} {(\text {L}{\rightarrow }^-)} \end{aligned}$$

then by the I.H. two tableaux each consisting of one of the premises are derivable in **bTKC**. Then we apply Lemma 19 to the tableau for the left premise to obtain the tableau $A{\rightarrow }B,\Gamma :\Delta \Rightarrow ^{*}C|A{\rightarrow }B,\Gamma :\Delta \Rightarrow ^{+} A$. The desired tableau is then obtained by (tL${\rightarrow }^-$). Other cases are analogous.

$$\square $$

#### Proposition 21

All rules of **bTKC** are depth-preserving invertible.

#### Proof

By induction on the depth of derivations. As an example, consider the invertibility of (tR${\rightarrow }^{-}$) and the last rule applied is an instance of (L${\rightarrow }^{-}$). If the sequent with respect to which the rule is applied does not have an implication in the succedent, then we can apply the I.H. to the premise tableau. Otherwise, we have:

$$\begin{aligned} \frac{\sigma |C{\rightarrow }D,\Gamma :\Delta \Rightarrow ^{-}A{\rightarrow }B|C{\rightarrow }D,\Gamma :\Delta \Rightarrow ^{+}C|\tau \qquad \sigma | D, C{\rightarrow }D,\Gamma :\Delta \Rightarrow ^{-}A{\rightarrow }B|\tau }{\sigma | C{\rightarrow }D,\Gamma :\Delta \Rightarrow ^{-}A{\rightarrow }B|\tau } {(\text {L}{\rightarrow }^{-})} \end{aligned}$$

Then by the I.H. the tableaux $\sigma |C{\rightarrow }D,\Gamma :\Delta ,A\Rightarrow ^{-}B|C{\rightarrow }D,\Gamma :\Delta \Rightarrow ^{+}C|\tau$ as well as $\sigma | D, C{\rightarrow }D,\Gamma :\Delta ,A\Rightarrow ^{-}B|\tau$ are derivable. Then we apply Lemma 19 to the former tableau to obtain $\sigma |C{\rightarrow }D,\Gamma :\Delta ,A\Rightarrow ^{-}B|C{\rightarrow }D,\Gamma :\Delta ,A\Rightarrow ^{+}C|\tau$. Thus we derive $\sigma | C{\rightarrow }D,\Gamma :\Delta ,A\Rightarrow ^{-}B|\tau$ by (L${\rightarrow }^{-}$). $\square$

We now establish that **bTKC** exhibits a better behaviour than **bGKC** when it comes to corresponding derivations for provable contradictions. The notions of correspondence of branches/derivations for tableaux are defined analogously to those for sequents, except that the signs of *all* sequents in a tableau have to be alternated.

#### Theorem 22

If $\textbf{bTKC}\vdash \ :\ \Rightarrow ^{+}A$ and $\textbf{bTKC}\vdash \ :\ \Rightarrow ^{-}A$, then there are derivations of the tableaux which correspond to each other.

#### Proof

The structure of the argument is identical to the argument given in the proof of Theorem 18. Hence we start with a derivation $d_{a}$ of $\ :\ \Rightarrow ^{+}A$, and first aim at constructing a pseudo-derivation $d'_{a}$ which corresponds to $d_{a}$. It must have a branch $t'_{0},\ldots ,t'_{n}$ that corresponds to a branch $t_{0},\ldots ,t_{n}$ of $d_{a}$, where $t'_{n}$ is a derivable initial tableau but may not be an instance of ($\text {tAx}^{-}$) or ($\text {tAx}^{+}$). In building $d'_{a}$ we proceed step by step: we assume at hand $t_{0},\ldots ,t_{i}$, $t'_{0},\ldots , t'_{i}$ as defined, as well as a pseudo-derivation $d'_{i}$ up to $t'_{i}$, and then define $t_{i+1}$, $t'_{i+1}$ and $d'_{i+1}$ depending on the rule applied to obtain $t_{i}$.

Let us denote by $\sigma ',\tau '$ the lists of sequents all of which have their sign alternated from $\sigma ,\tau$. If the rule is an instance of (tL${\rightarrow }^{-}$), e.g.:

$$\begin{aligned} \frac{\sigma |A{\rightarrow }B,\Gamma :\Delta \Rightarrow ^{+}C|A{\rightarrow }B,\Gamma :\Delta \Rightarrow ^{+}A|\tau \qquad \sigma | B, A{\rightarrow }B,\Gamma :\Delta \Rightarrow ^{+}C|\tau }{\sigma | A{\rightarrow }B,\Gamma :\Delta \Rightarrow ^{+}C|\tau } {(\text {tL}{\rightarrow }^{-})} \end{aligned}$$

then by the I.H. $t'_{i}$ is $\sigma '| A{\rightarrow }B,\Gamma :\Delta \Rightarrow ^{-}C|\tau '$. By Proposition 21, both tableaux $\sigma '|A{\rightarrow }B,\Gamma :\Delta \Rightarrow ^{-}C|A{\rightarrow }B,\Gamma :\Delta \Rightarrow ^{+}A|\tau '$ and $\sigma '| B, A{\rightarrow }B,\Gamma :\Delta \Rightarrow ^{-}C|\tau '$ are derivable: we define $t'_{i+1}$ to be the latter, $t_{i+1}$ to be the right premise of $t_{i}$, and define $d'_{i+1}$ by adding on top of $d'_{i}$ the following:

$$\begin{aligned} \frac{\sigma '|A{\rightarrow }B,\Gamma :\Delta \Rightarrow ^{-}C|A{\rightarrow }B,\Gamma :\Delta \Rightarrow ^{+}A|\tau ' \qquad \sigma '| B, A{\rightarrow }B,\Gamma :\Delta \Rightarrow ^{-}C|\tau '}{\sigma '| A{\rightarrow }B,\Gamma :\Delta \Rightarrow ^{-}C|\tau '} {(\text {tL}{\rightarrow }^{-})} \end{aligned}$$

where the derivation up to the left premise is arbitrarily taken: unlike in the case for **bGKC**, it cannot be copied from the left premise of $t_{i}$ due to the difference in signs.

If the rule is an instance of (tR$\wedge ^{-}$), e.g.:

$$\begin{aligned} \frac{\sigma |\Gamma :\Delta \Rightarrow ^{-}A\wedge B|\Gamma :\Delta \Rightarrow ^{-}B|\tau }{\sigma |\Gamma :\Delta \Rightarrow ^{-}A\wedge B|\tau } {(\text {tR}{\wedge }^-)} \end{aligned}$$

then by the I.H., $t'_{i}$ is $\sigma '|\Gamma :\Delta \Rightarrow ^{+}A\wedge B|\tau '$. By Proposition 21, both tableaux $\sigma '|\Gamma :\Delta \Rightarrow ^{+}A\wedge B|\Gamma :\Delta \Rightarrow ^{+}A|\tau '$ and $\sigma '|\Gamma :\Delta \Rightarrow ^{+}A\wedge B|\Gamma :\Delta \Rightarrow ^{+}B|\tau '$ are derivable. We define $t'_{i+1}$ to be the latter, $t_{i+1}$ to be the premise of $t_{i}$, and define $d'_{i+1}$ by adding on top of $d'_{i}$ the following:

$$\begin{aligned} \frac{\sigma '|\Gamma :\Delta \Rightarrow ^{+}A\wedge B|\Gamma :\Delta \Rightarrow ^{+}A|\tau ' \qquad \sigma '|\Gamma :\Delta \Rightarrow ^{+}A\wedge B|\Gamma :\Delta \Rightarrow ^{+}B|\tau '}{\sigma '|\Gamma :\Delta \Rightarrow ^{+}A\wedge B|\tau '} {(\text {tR}{\wedge }^+)} \end{aligned}$$

where the derivation up to the left premise is arbitrary taken.

If the rule is an instance of (tR$\wedge ^{+}$), e.g.:

$$\begin{aligned} \frac{\sigma |\Gamma :\Delta \Rightarrow ^{+}A\wedge B|\Gamma :\Delta \Rightarrow ^{+}A|\tau \qquad \sigma |\Gamma :\Delta \Rightarrow ^{+}A\wedge B|\Gamma :\Delta \Rightarrow ^{+}B|\tau }{\sigma |\Gamma :\Delta \Rightarrow ^{+}A\wedge B|\tau } {(\text {tR}{\wedge }^+)} \end{aligned}$$

then by the I.H., $t'_{i}$ is $\sigma '|\Gamma :\Delta \Rightarrow ^{-}A\wedge B|\tau '$. By Proposition 21, the tableau $\sigma '|\Gamma :\Delta \Rightarrow ^{-}A\wedge B|\Gamma :\Delta \Rightarrow ^{-}B|\tau '$ is derivable. We define $t'_{i+1}$ to be the tableau, $t_{i+1}$ to be the right premise of $t_{i}$, and define $d'_{i+1}$ by adding on top of $d'_{i}$ the following:

$$\begin{aligned} \frac{\sigma '|\Gamma :\Delta \Rightarrow ^{-}A\wedge B|\Gamma :\Delta \Rightarrow ^{-}B|\tau '}{\sigma |\Gamma :\Delta \Rightarrow ^{-}A\wedge B|\tau '} {(\text {tR}{\wedge }^-)} \end{aligned}$$

The other cases are similarly shown.

Having constructed $d'_{a}{:}{=}d'_{n}$, we choose a derivation $d'_{b}$ of $t'_{n}$ and construct another derivation $d_{b}$ of $t_{n}$ by the same process. As before, $d_{b}$ is in fact a derivation because $t_{n}$ is an instance of ($\text {tAx}^-$) or ($\text {tAx}^+$) and this is preserved along the branch we construct in defining $d_{b}$. Finally, define *d* ($d'$) by attaching $d_{b}$ ($d'_{b}$) on top of $d_{a}$ ($d'_{a}$): these derivations correspond to each other. $\square$

#### Example 2

As an example, we treat the case for the formula $((p\vee \mathord {\sim }p)\wedge (p\leftrightarrow \mathord {\sim }p))\rightarrow (p\vee \mathord {\sim }p)$, which has a disjunction occurring w.r.t $\mathcal {N}$ and so is outside the scope of Theorem 18. Suppose that we start from the next derivation verifying the formula: 
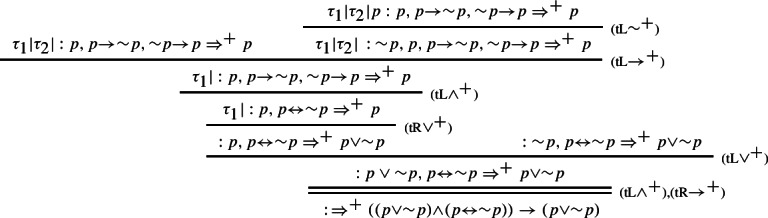


where $\tau _{1}$ is $\ :p,p{\leftrightarrow }\mathord {\sim }p\Rightarrow ^{+}p\vee \mathord {\sim }p$, $\tau _{2}$ is $\ :p,p{\rightarrow }\mathord {\sim }p,\mathord {\sim }p{\rightarrow }p\Rightarrow ^{+}p$ and the derivation above $\ :\mathord {\sim }p,p{\leftrightarrow }\mathord {\sim }p\Rightarrow ^{+}p\vee \mathord {\sim }p$ is abbreviated. Note that the first three lines of the derivation are in fact redundant: they are present to make the example simpler. If a derivation starts from the fourth line, then the first three lines may be added later to obtain a correspondence, following the procedure in the proof. In any case, we obtain the next derivation which falsifies the formula and has the middle branch corresponding to the middle branch in the above derivation, by applying the procedure: 
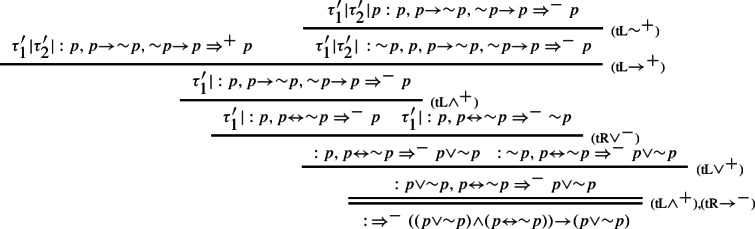


where $\tau _{1}$ is $\ :p,p{\leftrightarrow }\mathord {\sim }p\Rightarrow ^{-}p\vee \mathord {\sim }p$, $\tau _{2}$ is $\ :p,p{\rightarrow }\mathord {\sim }p,\mathord {\sim }p{\rightarrow }p\Rightarrow ^{-}p$ and the derivations above $\tau '_{1}|:p,p{\leftrightarrow }\mathord {\sim }p\Rightarrow ^{-}\mathord {\sim }p$ and $\ :\mathord {\sim }p,p{\leftrightarrow }\mathord {\sim }p\Rightarrow ^{-}p\vee \mathord {\sim }p$ are abbreviated.

### Analysis in terms of variables

Let us now come back to the topic of Subsection 3.2, namely of finding a property every derivation of a provable contradiction must satisfy. In the case of ${\textbf {bGKC}}_{\rightarrow }$, we saw that a contradictory formula must exhibit a sort of balance, and this property is inherited above along the rightmost branch. As we shall see, almost the same criterion can be adopted in the tableau setting of $\textbf{bTKC}$.

As additional preliminary notions, Definition 6 is expanded to $\mathcal {L}$ mostly in correspondence with Definition 9: so $pos^{+}(A\wedge B)= pos^{+}(A\vee B)=pos^{+}(A)\cup pos^{+}(B)$ and similarly for $pos^{+}_{\mathord {\sim }}$, $pos^{-}$, $pos^{-}_{\mathord {\sim }}$, *neg*, $neg_{\mathord {\sim }}$. We deviate from it in setting $spos(A\wedge B)=spos(A\vee B)=spos(A)\cap spos(B)$ and $spos_{\mathord {\sim }}(A\wedge B)=spos_{\mathord {\sim }}(A\vee B)=spos_{\mathord {\sim }}(A)\cap spos_{\mathord {\sim }}(B)$ for a reason we shall see in Theorem 24. Then we shall say a tableau is *balanced* if there is a sequent in the list which is balanced.

The next statement is different from Proposition 10 in that we just have the ‘only if’ direction, for which the preservation of balancedness holds for left premises as well.

#### Proposition 23

In $\textbf{bTKC}$, the conclusion of a rule is balanced only if its premises are.

#### Proof

By inspection of the rules. Here we treat the cases for (tL${\rightarrow }^{-}$); other cases follow analogously.

Consider an instance of (tL${\rightarrow }^-$):

$$\begin{aligned} \frac{\sigma |A{\rightarrow }B,\Gamma :\Delta \Rightarrow ^{+}C|A{\rightarrow }B,\Gamma :\Delta \Rightarrow ^{+} A|\tau \qquad \sigma |B,A{\rightarrow }B,\Gamma :\Delta \Rightarrow ^{+}C|\tau }{\sigma |A{\rightarrow }B,\Gamma :\Delta \Rightarrow ^{+}C|\tau } {(\text {tL}{\rightarrow }^-)} \end{aligned}$$

and assume that the conclusion is balanced. Then either $\sigma$ or $\tau$ has a sequent that is balanced, or $A{\rightarrow }B,\Gamma :\Delta \Rightarrow ^{+}C$ is balanced. In the first case, we can use the same sequent as a witness that each of the premises is balanced. In the second case, $A{\rightarrow }B,\Gamma :\Delta \Rightarrow ^{+}C$ can be used as a witness that the left premise is balanced, and we see by reusing the argument in the proof of Proposition 10 that $B,A{\rightarrow }B,\Gamma :\Delta \Rightarrow ^{+}C$ witnesses that the right premise is balanced.

$$\square $$

#### Theorem 24

If $\textbf{bTKC}\vdash \ \Rightarrow ^{+}A$ and $\textbf{bTKC}\vdash \ \Rightarrow ^{-}A$, then the tableaux are balanced.

#### Proof

The argument is as in Theorem 13. We need to check that Lemma 11 and 12 can be generalised for $\mathcal {L}$.

For Lemma 11 (i), we shall show $p\notin pos^{+}(A_{1}\circ A_{2})$ implies $\mathcal {M}^{p}_{1},w\Vdash ^{+}A_{1}\circ A_{2}$ for $\circ \in \{\wedge ,\vee \}$. As the assumptions we have for $i\in \{1,2\}$ that $p\notin pos^{+}(A_{i})$ implies $\mathcal {M}^{p}_{1},w\Vdash ^{+}A_{i}$. Hence the desired implication follows from the equivalence between $p\notin pos^{+}(A_{1}\circ A_{2})$ and ($p\notin pos^{+}(A_{1})$ and $p\notin pos^{+}(A_{2})$). The cases for negated conjunction and disjunction follow similarly, as well as the cases for Lemma 11 (ii).

For Lemma 12 (i), we shall show that $p\in spos(A\circ B)\cup spos_{\mathord {\sim }}(A\circ B)$ and $p\notin neg(A\circ B)$ implies $\mathcal {M}^{p}_{1},w\nVdash ^{+} A\circ B$ or $\mathcal {M}^{p}_{1},w\nVdash ^{-} A\circ B$, for $\circ \in \{\wedge ,\vee \}$. The first assumption implies^11^ either $p\in spos(A)\cap spos(B)$ or $p\in spos_{\mathord {\sim }}(A)\cap spos_{\mathord {\sim }}(B)$, and so we obtain $p\in spos(A)\cup spos_{\mathord {\sim }}(A)$ and $p\in spos(B)\cup spos_{\mathord {\sim }}(B)$ in both cases. In addition, the second assumption implies $p\notin neg(A)$ and $p\notin neg(B)$. Hence by the I.H. $\mathcal {M}^{p}_{1},w\nVdash ^{+} A$ or $\mathcal {M}^{p}_{1},w\nVdash ^{-} A$ as well as $\mathcal {M}^{p}_{1},w\nVdash ^{+} B$ or $\mathcal {M}^{p}_{1},w\nVdash ^{-} B$. Consequently, either $\mathcal {M}^{p}_{1},w\nVdash ^{+} A\circ B$ or $\mathcal {M}^{p}_{1},w\nVdash ^{-} A\circ B$ for $\circ \in \{\wedge ,\vee \}$. The case for Lemma 12 (ii) is analogous.

$$\square $$

#### Corollary 25

If a construction of a derivation of **bTKC** begins with an instance of ($\text {tAx}^{-}$) or ($\text {tAx}^{+}$) that is not balanced, then it never derives a provable contradiction.

#### Proof

Suppose that the construction ends in a derivation of a provable contradiction. Then the tableau is balanced by Theorem 24, but all tableaux in the derivation must then be balanced by Proposition 23, a contradiction. $\square$

## Preliminaries for the predicate case

From now on, we shall employ a predicate language $\mathcal {L}_{q}$ in order to investigate the predicate logic **QC** [22]. It has a countably infinite supply $\mathcal {P}^{n}_{1},P^{n}_{2},\ldots$ of *n*-ary *predicate symbols* for each $n\in \mathbb {N}$; countably infinite supplies $v_{1},v_{2},\ldots$ of *variables* and $c_{1},c_{2},\ldots$ of *constants*. A *term* is either a variable or a constant. As metavariables, we shall use $P,Q,\ldots$ for predicates, $x,y,\ldots$ for variables, $c,d,\ldots$ for constants, and $s,t,\ldots$ for terms. Formulas in $\mathcal {L}_{q}$ are defined by the next clause:

$$\begin{aligned} A {::}{=} P(t_{1},\ldots , t_{n})\ |\ (A\wedge A)\ |\ (A\vee A)\ |\ (A\rightarrow A)\ |\ \mathord {\sim }A\ |\ \forall {x}A\ |\ \exists {x}A. \end{aligned}$$

(where *P* is *n*-ary). The notions of substitution, free and bound variables are defined as usual: see e.g. [21, pp.3-5]. The result of a substitution of a term *t* for a variable *x* in an expression $\alpha$ will be denoted by $\alpha [x/t]$. Two formulas are taken to be identical if they differ only in the names of bound variables, and a substitution is assumed to involve a renaming of bound variables when necessary.

### Proof theory

We next introduce proof systems for the predicate expansion of **C**, starting with a Hilbert-style system introduced in [22].

#### Definition 11

The system **QC** is defined in $\mathcal {L}_{q}$ by the addition of the next axiom schemata and rules to **C**.

A13 $$\begin{aligned} A[x/t]\rightarrow \exists {x}A \end{aligned}$$

A14 $$\begin{aligned} \forall {x}A\rightarrow A[x/t] \end{aligned}$$

P1 $$\begin{aligned} {\frac{A\rightarrow B[x/y]}{A\rightarrow \forall {x}B}} \end{aligned}$$

A15 $$\begin{aligned} \mathord {\sim }\forall {x}A\leftrightarrow \exists {x}\mathord {\sim }A \end{aligned}$$

A16 $$\begin{aligned} \mathord {\sim }\exists {x}A\leftrightarrow \forall {x}\mathord {\sim }A \end{aligned}$$

P2 $$\begin{aligned} {\frac{B[x/y]\rightarrow A}{\exists {x}B\rightarrow A}} \end{aligned}$$

where *y* is not free in *A* in (P1) and (P2). A *derivation* of a formula is defined as in Definition 1 but incorporating the new rules.

The sequent calculus **bGKC** is also modified to include rules for the quantifiers.

#### Definition 12

The sequent calculus **bGKQC** is defined from **bGKC** by replacing the rules ($\text {Ax}^-$) and ($\text {Ax}^+$) with the following rules (where $P$ is an atomic formula.).

$$\begin{aligned} P,\Gamma :\Delta \Rightarrow ^{-} P (qAx^-)&\Gamma :\Delta ,P\Rightarrow ^{+} P (qAx^+) \\ \frac{A[x/y],\Gamma :\Delta \Rightarrow ^{*}C}{\forall {x}A,\Gamma :\Delta \Rightarrow ^{*}C} {(L\forall ^{-})}&\frac{\Gamma :\Delta \Rightarrow ^{-}A[x/t]}{\Gamma :\Delta \Rightarrow ^{-}\forall {x}A} {(R\forall ^{-})} \\ \frac{\Gamma :\Delta ,A[x/t],\forall {x}A\Rightarrow ^{*}C}{\Gamma :\Delta ,\forall {x}A\Rightarrow ^{*}C} {(L\forall ^{+})}&\frac{\Gamma :\Delta \Rightarrow ^{+}A[x/y]}{\Gamma :\Delta \Rightarrow ^{+}\forall {x}A} {(R\forall ^{+})} \\ \frac{\exists {x}A,A[x/t],\Gamma :\Delta \Rightarrow ^{*}C}{\exists {x}A,\Gamma :\Delta \Rightarrow ^{*}C} {(L\exists ^{-})}&\frac{\Gamma :\Delta \Rightarrow ^{-}A[x/y]}{\Gamma :\Delta \Rightarrow ^{-}\exists {x}A} {(R\exists ^{-})} \\ \frac{\Gamma :\Delta ,A[x/y]\Rightarrow ^{*}C}{\Gamma :\Delta ,\exists {x}A\Rightarrow ^{*}C} {(L\exists ^{+})}&\frac{\Gamma :\Delta \Rightarrow ^{+}A[x/t]}{\Gamma :\Delta \Rightarrow ^{+}\exists {x}A} {(R\exists ^{+})} \end{aligned}$$

where *y* does not occur free in the conclusion in (L$\forall ^{-}$), (R$\forall ^{+}$), (R$\exists ^{-}$) and (L$\exists ^{+}$).

Proposition 1 can be generalised to $\mathcal {L}_{q}$ in this calculus.

#### Proposition 26

$$\textbf{bGKQC}\vdash A,\Gamma :\Delta \Rightarrow ^{-} A$$ and $$\textbf{bGKQC}\vdash \Gamma :\Delta ,A\Rightarrow ^{+} A$$.

#### Proof

It suffices to check the cases for quantifiers. For the universal quantifier, take a variable *y* which does not occur free in $\forall {x}A$, $\Gamma$ or $\Delta$. Then: 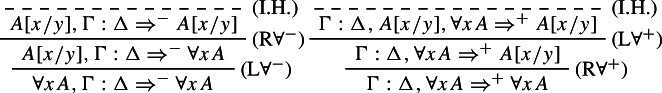 The case for $\exists$ is analogous. $\square$

We shall next check some standard properties needed to establish cut-elimination.

#### Lemma 27

If $\textbf{bGKQC}\vdash _{k} \Gamma :\Delta \Rightarrow ^{*}C$ then $\textbf{bGKQC}\vdash _{k} \Gamma ':\Delta '\Rightarrow ^{*}C'$ where $\Gamma '$, $\Delta '$ and $C'$ are obtained by replacing bound variables with fresh ones.

#### Proof

We show the claim by induction on the depth of derivations. If a derivation ends with an instance of ($\text {qAx}^-$) or ($\text {qAx}^+$), then all bound variables occur in the contexts, so the statement holds by another instance of ($\text {qAx}^-$) or ($\text {qAx}^+$). The cases for propositional connectives follow straightforwardly from the inductive hypothesis.

If a derivation (of depth $k+1$) ends with an instance of (L$\forall ^{-}$):

$$\begin{aligned} \frac{A[x/y],\Gamma :\Delta \Rightarrow ^{*}C}{\forall {x}A,\Gamma :\Delta \Rightarrow ^{*}C} \end{aligned}$$

We want to show $\vdash _{k+1}\forall {z}(A'[x/z]),\Gamma ':\Delta '\Rightarrow ^{*}C'$ where bound variables are replaced with fresh ones, and in particular *x* is replaced with *z*. By the I.H. $\vdash _{k} A'[x/z][z/y],\Gamma ':\Delta '\Rightarrow ^{*}C'$ and so we obtain $\vdash _{k+1} \forall {z}(A'[x/z]),\Gamma ':\Delta '\Rightarrow ^{*}C'$ by (L$\forall ^{-}$).

If a derivation (of depth $k+1$) ends with an instance of (L$\forall ^{+}$):

$$\begin{aligned} \frac{\Gamma :\Delta ,A[x/t],\forall {x}A\Rightarrow ^{*}C}{\Gamma :\Delta ,\forall {x}A\Rightarrow ^{*}C} \end{aligned}$$

We want to show $\vdash _{k+1}\Gamma ':\Delta ',\forall {z}(A'[x/z])\Rightarrow ^{*}C'$. By the I.H. we obtain that $\vdash _{k}\Gamma ':\Delta ',A'[x/z][z/t],\forall {z}(A'[x/z])\Rightarrow ^{*}C'$. Thus the desired sequent follows by (L$\forall ^{+}$).

$$\square $$

#### Lemma 28

If $\textbf{bGKQC}\vdash _{k} \Gamma :\Delta \Rightarrow ^{*}C$ then $\textbf{bGKQC}\vdash _{k}\Gamma [x/t]:\Delta [x/t]\Rightarrow ^{*}C[x/t]$ where *t* is free for *x* in $\Gamma$, $\Delta$ and *C*.

#### Proof

We show the claim by induction on the depth of derivations. For ($\text {qAx}^{-}$), we have $P,\Gamma :\Delta \Rightarrow ^{-}P$. Suppose *t* is free for *x* in the sequent. Then $P[x/t],\Gamma [x/t]:\Delta [x/t]\Rightarrow ^{-}P[x/t]$ is another instance. The case for ($\text {qAx}^{+}$) is similarly argued. The cases for propositional connectives follow straightforwardly from the I.H..

Suppose the last step in the derivation (of depth $k+1$) is an instance of (L$\forall ^{-}$):

$$\begin{aligned} \frac{A[y/z],\Gamma :\Delta \Rightarrow ^{*}C}{\forall {y}A,\Gamma :\Delta \Rightarrow ^{*}C} \end{aligned}$$

where *z* is not free in the conclusion. Assume *t* is free for *x* in the conclusion. We divide into cases depending on whether $x=y$: here we check the case when $x\ne y$.

If $x\ne y$, then our goal is to show $\vdash _{k+1}(\forall {y}A)[x/t],\Gamma [x/t]:\Delta [x/t]\Rightarrow ^{*}C[x/t]$. By the I.H.

$$\begin{aligned} \vdash _{k}A[y/z][z/z'],\Gamma :\Delta \Rightarrow ^{*}C \end{aligned}$$

where $z'$ is fresh. Then *t* is free for *x* in $A[y/z][z/z']$. Apply the I.H. again to obtain:

$$\begin{aligned} \vdash _{k}A[y/z][z/z'][x/t],\Gamma [x/t]:\Delta [x/t]\Rightarrow ^{*}C[x/t]. \end{aligned}$$

Now we have $A[y/z][z/z'][x/t]=A[x/t][y/z][z/z']=A[x/t][y/z']$ irrespective of whether *x* occurs free in $\forall {y}A$, because *t* is free for *x* in $\forall {y}A$. Therefore by (L$\forall ^{-}$) we obtain

$$\begin{aligned} \vdash _{k+1}(\forall {y}A)[x/t],\Gamma [x/t]:\Delta [x/t]\Rightarrow ^{*}C[x/t] \end{aligned}$$

as $$\forall {y}(A[x/t])=(\forall {y}A)[x/t]$$.

Suppose the last step in the derivation (of depth $k+1$) is an instance of (L$\forall ^{+}$):

$$\begin{aligned} \frac{\Gamma :\Delta ,A[y/s],\forall {y}A\Rightarrow ^{*}C}{\Gamma :\Delta ,\forall {y}{A}\Rightarrow ^{*}C} \end{aligned}$$

Assume *t* is free for *x* in the conclusion. Again, let us look at the case $x\ne y$.

If $x\ne y$, then we want to establish that $\vdash _{k+1}\Gamma [x/t]:\Delta [x/t],(\forall {y}{A})[x/t]\Rightarrow ^{*}C[x/t]$. By Lemma 27, we have:

$$\begin{aligned} \vdash _{k}\Gamma ':\Delta ',A'[y/y'][y'/s],\forall {y'}(A'[y/y'])\Rightarrow ^{*}C' \end{aligned}$$

where $y'$ is fresh and $\Gamma '$, $\Delta '$, $A'$ and $C'$ are obtained by replacing bound variables with fresh ones. Then *t* is free for *x* in the sequent. Consequently,

$$\begin{aligned} \vdash _{k}\Gamma '[x/t]:\Delta '[x/t],A'[y/y'][y'/s][x/t],(\forall {y'}(A'[y/y']))[x/t]\Rightarrow ^{*}C'[x/t]. \end{aligned}$$

As *t* is free for *x* in $\forall {y}A$ and $x\ne y$, this sequent can be re-expressed as:

$$\begin{aligned} \vdash _{k}\Gamma '[x/t]:\Delta '[x/t],A'[x/t][y/y'][y'/s[x/t]],(\forall {y'}(A'[x/t][y/y']))\Rightarrow ^{*}C'[x/t]. \end{aligned}$$

Thus by (L$\forall ^{+}$) and $\forall {y'}(A'[x/t][y/y'])=\forall {y'}(A'[y/y'])[x/t]$, it follows that

$$\begin{aligned} \vdash _{k+1}\Gamma '[x/t]:\Delta '[x/t],\forall {y'}(A'[y/y'])[x/t]\Rightarrow ^{*}C'[x/t] \end{aligned}$$

which is identified with the target sequent. Other cases are analogous.

$$\square $$

#### Proposition 29

($\text {LW}^{-}$) and ($\text {LW}^{+}$) are depth-preserving admissible in **bGKQC**.

#### Proof

By induction on the depth of derivations. Here we give an example for ($\text {LW}^{-}$) when the depth is $k+1$ and the last rule applied is an instance of (R$\forall ^{+}$).

$$\begin{aligned} \frac{\Gamma :\Delta \Rightarrow ^{+}C[x/y]}{\Gamma :\Delta \Rightarrow ^{+}\forall {x}C} {(\text {R}\forall ^{+})} \end{aligned}$$

where *y* does not occur free in the conclusion. Then $\vdash _{k}\Gamma :\Delta \Rightarrow ^{+}C[x/y][y/y']$ for $y'$ fresh, by Lemma 28. By the I.H. this implies $\vdash _{k}A, \Gamma :\Delta \Rightarrow ^{+}C[x/y][y/y']$. Now we may apply (R$\forall ^{+}$), and so $\vdash _{k+1}A, \Gamma :\Delta \Rightarrow ^{+}\forall {x}C$. $\square$

It can be checked straightforwardly that Lemma 3 holds in **bGKQC** as well. Moreover, we have the next invertibility for quantifier rules.

#### Lemma 30

The following statements hold in **bGKQC**.

(i) If $$\vdash _{k}\forall {x}A,\Gamma :\Delta \Rightarrow ^{*}C$$ then $$\vdash _{k}A[x/y],\Gamma :\Delta \Rightarrow ^{*}C$$.
(ii) If $\vdash _{k}\Gamma :\Delta \Rightarrow ^{+}\forall {x}A$ then $\vdash _{k}\Gamma :\Delta \Rightarrow ^{+}A[x/y]$.
(iii) If $\vdash _{k}\Gamma :\Delta ,\exists {x}A\Rightarrow ^{*} C$ then $\vdash _{k}\Gamma :\Delta ,A[x/y]\Rightarrow ^{*}C$.
(iv) If $\vdash _{k}\Gamma :\Delta \Rightarrow ^{-}\exists {x} A$ then $\vdash _{k}\Gamma :\Delta \Rightarrow ^{-}A[x/y]$.

where *y* does not occur free in $\Gamma$, $\Delta$, *A* nor in *C*.

#### Proof

By induction on the depth of derivations. Here we look at (iii), where the depth of derivations is $k+1$ and the last rule applied is an instance of (L$\exists ^{+}$).

$$\begin{aligned} \frac{\Gamma :\Delta ,A[x/z]\Rightarrow ^{*}C}{\Gamma :\Delta ,\exists {x}A\Rightarrow ^{*}C} {(\text {L}\exists ^{+})} \end{aligned}$$

where *z* does not occur free in the conclusion. By Lemma 28, it holds that $\vdash _{k}\Gamma :\Delta ,A[x/z][z/y]\Rightarrow ^{*}C$.

$$\square $$

#### Lemma 31

($\text {LC}^{-}$) and ($\text {LC}^{+}$) are depth-preserving admissible in **bGKQC**.

#### Proof

By induction on the depth of derivations. Consider e.g. the case for ($\text {LC}^-$) where the depth of derivations is $k+1$, the formula in question is an existential formula which is obtained through an application of (L$\exists ^{-}$):

$$\begin{aligned} \frac{\exists {x}A,A[x/t],\exists {x}A,\Gamma :\Delta \Rightarrow ^{*}C}{\exists {x}A,\exists {x}A,\Gamma :\Delta \Rightarrow ^{*}C} \end{aligned}$$

Then apply the I.H. to the premise and then apply (L$\exists ^{-}$). $\square$

We are now ready to establish the cut-elimination for **bGKQC**.

#### Theorem 32

($\text {Cut}^{-}$) and ($\text {Cut}^+$) are admissible in **bGKQC**.

#### Proof

The general outline is identical to Theorem 6. Here we look at the case of ($\text {Cut}^-$) where the cutformula is principal in both premises, and is a universal formula. 
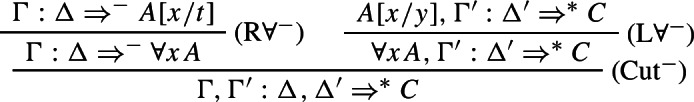
 where *y* does not occur free in $\forall {x}A$, $\Gamma '$, $\Delta '$ and *C*. Then *t* is free for *y* in *A*[*x*/*y*], $\Gamma '$,$\Delta '$ and *C*. Thus we obtain the following derivation of less complexity. 
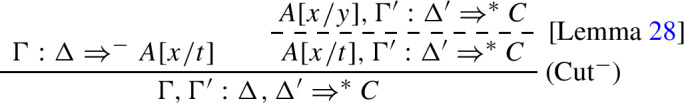
$$\square $$

#### Corollary 33

$\textbf{bGKQC}\vdash \Gamma :\Delta \Rightarrow ^{*}A$ if and only if $\textbf{QC}\vdash \bigwedge \Gamma ^{-}\wedge \bigwedge \Delta ^{+}\rightarrow A^{*}$.

#### Proof

The argument is analogous to Corollary 7. $\square$

Our next step is the introduction of a tableau calculus for **QC**.

#### Definition 13

The tableau calculus **bTKQC**, is obtained from **bGKQC** in the same way **bTKC** is defined from **bGKC**.^12^ In particular, ($\text {qAx}^-$) and ($\text {qAx}^+$) are turned into rules (where $P$ is an atomic formula):

$$\begin{aligned} \sigma |P,\Gamma :\Delta \Rightarrow ^{-} P|\tau (tqAx^-)\qquad \sigma |\Gamma :\Delta ,P\Rightarrow ^{+} P|\tau (tqAx^+) \end{aligned}$$

For the quantifiers, the right rules are modified in the following way.

$$\begin{aligned} \frac{\sigma |\Gamma :\Delta \Rightarrow ^{-}\forall {x}A|\Gamma :\Delta \Rightarrow ^{-}A[x/t]|\tau }{\sigma |\Gamma :\Delta \Rightarrow ^{-}\forall {x}A|\tau } {(tR\forall ^{-})}  &   \frac{\sigma |\Gamma :\Delta \Rightarrow ^{+}\forall {x}A|\Gamma :\Delta \Rightarrow ^{+}A[x/y]|\tau }{\sigma |\Gamma :\Delta \Rightarrow ^{+}\forall {x}A|\tau } {(tR\forall ^{+})} \\ \frac{\sigma |\Gamma :\Delta \Rightarrow ^{-}\exists {x}A|\Gamma :\Delta \Rightarrow ^{-}A[x/y]|\tau }{\sigma |\Gamma :\Delta \Rightarrow ^{-}\exists {x}A|\tau } {(tR\exists ^{-})}  &   \frac{\sigma |\Gamma :\Delta \Rightarrow ^{+}\exists {x}A|\Gamma :\Delta \Rightarrow ^{+}A[x/t]|\tau }{\sigma |\Gamma :\Delta \Rightarrow ^{+}\exists {x}A|\tau } {(tR\exists ^{+})} \end{aligned}$$

where *y* does not occur free in the conclusion in (tR$\forall ^{+}$) and (tR$\exists ^{-}$).

We now observe some properties of this tableau calculus.

#### Lemma 34

The following statements hold.

(i) If $\textbf{bTKQC}\vdash _{k} \sigma |\Gamma :\Delta \Rightarrow ^{*}C|\tau$ then $\textbf{bGKQC}\vdash _{k} \sigma '|\Gamma ':\Delta '\Rightarrow ^{*}C'|\tau '$ where $\sigma '$, $\Gamma '$, $\Delta '$, $C'$ and $\tau '$ are obtained by replacing bound variables with fresh ones.
(ii) Assume $\textbf{bTKQC}\vdash \sigma |\Gamma :\Delta \Rightarrow ^{*}C|\tau$ and *t* is free for *x* in $\sigma$,$\Gamma$,$\Delta$, *C*, $\tau$. Then $\textbf{bTKQC}\vdash \sigma [x/t]|\Gamma [x/t]:\Delta [x/t]\Rightarrow ^{*}C[x/t]|\tau [x/t]$.

#### Proof

Analogous to Lemma 27, 28.

$$\square $$

#### Lemma 35

($\text {tLW}^{-}$), ($\text {tLW}^{+}$) and (tW) are depth-preserving admissible in **bTKQC**.

#### Proof

It is proved similarly to Proposition 29 using Lemma 34.

$$\square $$

#### Proposition 36

$\textbf{bTGQC}\vdash \Gamma :\Delta \Rightarrow ^{*}C$ if and only if $\textbf{bGKQC}\vdash \Gamma :\Delta \Rightarrow ^{*}C$.

#### Proof

The argument follows that of Proposition 20 in both directions. For the right-to-left direction, we appeal to Lemma 35.

$$\square $$

#### Proposition 37

All rules of **bTKQC** are depth-preserving invertible.

#### Proof

By induction on the depth of derivations. Here we check the cases for quantifiers. The cases for the right rules are immediate. For the cases of the left rules, consider a case for (tL$\forall ^-$) in which the derivation is of depth $k+1$ and the last rule applied is an instance of (tR$\forall ^{-}$):

$$\begin{aligned} \frac{\sigma |\forall {x}A,\Gamma :\Delta \Rightarrow ^{-}\forall {y}B|\forall {x}A,\Gamma :\Delta \Rightarrow ^{-}B[y/t]|\tau }{\sigma |\forall {x}A,\Gamma :\Delta \Rightarrow ^{-}\forall {y}B|\tau } {(\text {tR}\forall ^{-})} \end{aligned}$$

Take *z* which does not occur free in the conclusion. We need to show:

$$\begin{aligned} \vdash _{k+1}\sigma |A[x/z],\Gamma :\Delta \Rightarrow ^{-}\forall {y}B|\tau . \end{aligned}$$

Towards this, first we use Lemma 34 (ii) to obtain (for *u* fresh):

$$\begin{aligned} \vdash _{k}\sigma |\forall {x}A,\Gamma :\Delta \Rightarrow ^{-}\forall {y}B|\forall {x}A,\Gamma :\Delta \Rightarrow ^{-}B[y/t[z/u]]|\tau . \end{aligned}$$

Now we can apply the I.H. to obtain:

$$\begin{aligned} \vdash _{k}\sigma |A[x/z],\Gamma :\Delta \Rightarrow ^{-}\forall {y}B|\forall {x}A,\Gamma :\Delta \Rightarrow ^{-}B[y/t[z/u]]|\tau . \end{aligned}$$

Let *v* be another fresh variable. We apply the I.H. once more to obtain

$$\begin{aligned} \vdash _{k}\sigma |A[x/z],\Gamma :\Delta \Rightarrow ^{-}\forall {y}B|A[x/v],\Gamma :\Delta \Rightarrow ^{-}B[y/t[z/u]]|\tau . \end{aligned}$$

Apply next Lemma 34 (ii) again to infer:

$$\begin{aligned} \vdash _{k}\sigma |A[x/z],\Gamma :\Delta \Rightarrow ^{-}\forall {y}B|A[x/v][v/z],\Gamma :\Delta \Rightarrow ^{-}B[y/t[z/u]]|\tau . \end{aligned}$$

The desired tableau is now obtained by (tR$\forall ^{-}$). Other cases are similarly argued. $\square$

### Semantics

Turning our attention to the semantical side again, we introduce Kripke semantics for **QC**. Such semantics have already been introduced in [18] and [22] in slightly different ways. The exposition here is based on the latter.

#### Definition 14

A *Kripke frame* $\mathcal {F}$ for **QC** is defined as before. A *Kripke model* for **QC** is a quintuple $(\mathcal {F},\Delta ,\delta ,\mathcal {V}^{+},\mathcal {V}^{-})$ where $\mathcal {F}=(W,\le )$ is a Kripke frame; $\Delta$ is a set of terms in $\mathcal {L}_{q}$ containing all constants; $\delta :W\rightarrow \mathcal {P}(\Delta )$ is such that $\delta (w)$ (the *domain* of *w*) contains all constants; $\mathcal {V}^{+}$ and $\mathcal {V}^{-}$ respectively assigns a set $\mathcal {V}^{+}(P(t_{1},\ldots ,t_{n}))$ and $\mathcal {V}^{-}(P(t_{1},\ldots ,t_{n}))$ for each atomic formula $P(t_{1},\ldots ,t_{n})$ with $t_{1},\ldots ,t_{n}\in \Delta$. A Kripke model has to further satisfy the next conditions.

1. If $$w'\ge w$$ then $$\delta (w')\supseteq \delta (w)$$.
2. If $$w\in \mathcal {V}^{*}(P(t_{1},\ldots ,t_{n}))$$ then $$t_{1},\ldots , t_{n}\in \delta (w)$$.
3. If $w\in \mathcal {V}^{*}(P(t_{1},\ldots ,t_{n}))$ and $w'\ge w$ then $w'\in \mathcal {V}^{*}(P(t_{1},\ldots , t_{n}))$.

$\mathcal {V}^{+}$ and $\mathcal {V}^{-}$ are each extended to forcing at a world *w* of compound sentences in $\delta (w)$. 
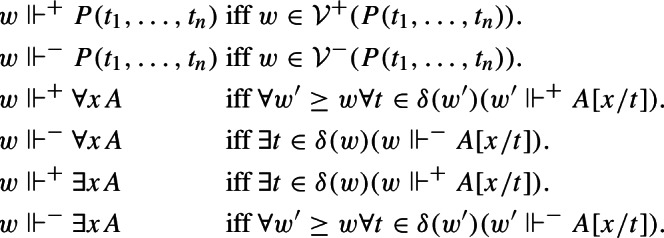
 For a formula *A*, we write $\vDash A$ if $\mathcal {M},w\Vdash ^{+}A$ for all *w* in any model $\mathcal {M}$.

#### Proposition 38

$\textbf{bTKQC}\vdash \ :\ \Rightarrow ^{+} A$ if and only if $\textbf{QC}\vDash A$.

#### Proof

Given Corollary 33, it suffices to check the soundness and the completeness with respect to **QC**, which is established in [22]. $\square$

## Provable Contradictions in **bTKQC**

In studying the relationship between verification and falsification of a formula in **bGKQC**, we shall expand the notion of correspondence to the predicate language in a liberal way: a pair $s_{i}$ and $s'_{i}$ of sequents in corresponding branches need not have identical formulas. We allow differences in non-bound terms such as the one between $\Gamma :\Delta \Rightarrow ^{+}A[x/c]$ and $\Gamma :\Delta \Rightarrow ^{-}A[x/y]$. This makes it possible to consider (tR$\forall ^{-}$) and (tR$\forall ^{+}$) for instance (note the difference in both the signs and the substituting terms) to be preserving a correspondence.

### Theorem 39

If $\textbf{bTKQC}\vdash \ :\ \Rightarrow ^{+}A$ and $\textbf{bTKQC}\vdash \ :\ \Rightarrow ^{-}A$, then there are derivations of the tableaux which correspond to each other.

### Proof

The outline of the proof follows that of the proof of Theorem 22. So consider a derivation $d_{a}$ of $\ :\ \Rightarrow ^{+}A$ and assume that a pseudo-derivation $d'_{i}$ with a branch $t'_{0},\ldots , t'_{i}$ is defined, in such a way that a branch $t_{0}\ldots ,t_{i}$ (of the pseudo-derivation defined by removing tableaux above $t_{i}$ from $d_{a}$) corresponds to it. We must choose $t_{i+1}$ and $t'_{i+1}$ that preserve the correspondence. As before, we divide into cases depending on which rule is applied to obtain $t_{i}$. The only differences from Theorem 22 are the cases where it is obtained by one of the quantifier rules.

If the rule applied to obtain $t_{i}$ is an instance of (tL$\forall ^{-}$), e.g.:

$$\begin{aligned} \frac{\sigma |B[x/y],\Gamma :\Delta \Rightarrow ^{+}C|\tau }{ \sigma |\forall {x}B,\Gamma :\Delta \Rightarrow ^{+}C|\tau } {(\text {tL}\forall ^{-})} \end{aligned}$$

where *y* does not occur free in the conclusion. Then we choose $t_{i+1}$ to be the upper tableau. By the I.H., $t'_{i+1}$ has the form $\sigma '|\forall {x}B',\Gamma ':\Delta '\Rightarrow ^{-}C'|\tau '$ where $\sigma '$, $\forall {x}B'$, $\Gamma '$, $\Delta '$, $C'$ and $\tau '$ are identical to $\sigma$, $\forall {x}B$, $\Gamma$, $\Delta$, *C* and $\tau$ except for non-bound terms (a similar convention is applied in subsequent cases). By Proposition 37, $\sigma '|B'[x/z],\Gamma ':\Delta '\Rightarrow ^{-}C'|\tau '$ is derivable for a fresh *z*. Take this tableau as $t'_{i+1}$ and define $d'_{i+1}$ by adding on top of $d'_{i}$ the following step:

$$\begin{aligned} \frac{\sigma '|B'[x/z],\Gamma ':\Delta '\Rightarrow ^{-}C'|\tau '}{\sigma '|\forall {x}B',\Gamma ':\Delta '\Rightarrow ^{-}C'|\tau '} {(\text {tL}\forall ^{-})} \end{aligned}$$

If the rule applied to obtain $t_{i}$ is an instance of (tR$\forall ^{-}$), e.g.:

$$\begin{aligned} \frac{\sigma |\Gamma :\Delta \Rightarrow ^{-}\forall {x}B|\Gamma :\Delta \Rightarrow ^{-}B[x/t]|\tau }{\sigma |\Gamma :\Delta \Rightarrow ^{-}\forall {x}B|\tau } {(\text {tR}\forall ^{-})} \end{aligned}$$

Then choose the upper tableau as $t_{i+1}$. By the I.H., $t'_{i}$ has the form $\sigma '|\Gamma ':\Delta '\Rightarrow ^{+}\forall {x}B'|\tau '$. By Lemma 35, $\sigma '|\Gamma ':\Delta '\Rightarrow ^{+}\forall {x}B'|\Gamma ':\Delta '\Rightarrow ^{+}B'[x/y]|\tau '$ is derivable for a fresh *y*. Choose it as $t'_{i+1}$, and $d'_{i+1}$ is obtained by adding on top of $d'_{i}$ the next step:

$$\begin{aligned} \frac{\sigma '|\Gamma ':\Delta '\Rightarrow ^{+}\forall {x}B'|\Gamma ':\Delta '\Rightarrow ^{+}B'[x/y]|\tau '}{\sigma '|\Gamma ':\Delta '\Rightarrow ^{+}\forall {x}B'|\tau '} {(\text {tR}\forall ^{+})} \end{aligned}$$

If the rule applied to obtain $t_{i}$ is an instance of (tL$\forall ^+$), e.g.:

$$\begin{aligned} \frac{\sigma |\Gamma :\Delta ,B[x/t],\forall {x}B\Rightarrow ^{+}C|\tau }{\sigma |\Gamma :\Delta ,\forall {x}B\Rightarrow ^{+}C|\tau } {(\text {tL}\forall ^{+})} \end{aligned}$$

then choose the upper tableau as $t_{i+1}$. By the I.H. $t'_{i}$ has the form $\sigma '|\Gamma ':\Delta ',\forall {x}B'\Rightarrow ^{-}C'|\tau '$. By Lemma 35, we obtain $\sigma '|\Gamma ':\Delta ', B'[x/t],\forall {x}B'\Rightarrow ^{-}C'|\tau '$. Choose this tableau as $t'_{i+1}$ and define $d'_{i+1}$ by attaching the next step on top of $d'_{i}$:

$$\begin{aligned} \frac{\sigma '|\Gamma ':\Delta ',B'[x/t],\forall {x}B'\Rightarrow ^{-}C'|\tau '}{\sigma '|\Gamma ':\Delta ',\forall {x}B'\Rightarrow ^{-}C'|\tau '} {(\text {tL}\forall ^{+})} \end{aligned}$$

If the rule applied to obtain $t_{i}$ is an instance of (tR$\forall ^{+}$), e.g.:

$$\begin{aligned} \frac{\sigma |\Gamma :\Delta \Rightarrow ^{+}\forall {x}B|\Gamma :\Delta \Rightarrow ^{+}B[x/y]|\tau }{\sigma |\Gamma :\Delta \Rightarrow ^{+}\forall {x}B|\tau } {(\text {tR}\forall ^{+})} \end{aligned}$$

where *y* is free in the conclusion. Choose the upper tableau as $t_{i+1}$. By the I.H., $t'_{i}$ is $\sigma '|\Gamma ':\Delta '\Rightarrow ^{-}\forall {x}B'|\tau '$. Then $\sigma '|\Gamma ':\Delta '\Rightarrow ^{-}\forall {x}B'|\Gamma ':\Delta '\Rightarrow ^{-}B'[x/y]|\tau '$ is derivable from Lemma 35, so we take it as $t'_{i+1}$. Define $d'_{i+1}$ by attaching the next step to $d'$:

$$\begin{aligned} \frac{\sigma '|\Gamma ':\Delta '\Rightarrow ^{-}\forall {x}B'|\Gamma ':\Delta '\Rightarrow ^{-}B'[x/y]|\tau '}{\sigma '|\Gamma ':\Delta '\Rightarrow ^{+}\forall {x}B'|\tau '} {(\text {tR}\forall ^{-})} \end{aligned}$$

The cases of the rules for the existential quantifier follow in a similar manner.

We now have $d_{a}$ and a pseudo-derivation $d'_{a}{:}{=}d'_{n}$ corresponding to it, such that $t_{n}$ has e.g. the form $\sigma |\Gamma :\Delta ,P\Rightarrow ^{+}P|\tau$ and $t'_{n}$ has the form $\sigma '|\Gamma ':\Delta ',P'\Rightarrow ^{-}P''|\tau '$. The latter tableau may not be an initial tableau, but is derivable. Thus we can repeat the same process with respect to derivations of $t'_{n}$ and construct a derivation of $t_{n}$ which corresponds to that derivation. Since no rule affects atomic formulas, the last tableau in the corresponding branch starting from $t_{n}$ is another instance of an initial tableau, in this case ($\text {tqAx}^{+}$). The rest follows as in Theorem 22.

$$\square $$

### Example 3

Consider the following derivation falsifying $(\forall {x}P(x)\wedge \mathord {\sim }P(c))\rightarrow \forall {x}P(x)$, a provable contradiction in **QC**:
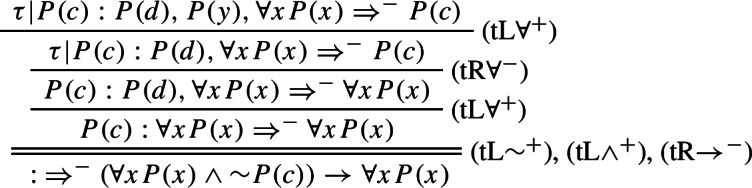
 where $\tau$ is $P(c):P(d),\forall {x}P(x)\Rightarrow ^{-}\forall {x}P(x)$ and *y* does not occur free in the third line. Note that the first line is redundant, but if we start from the second line then the first line has to be added up for a correspondence: as in Example 2, we use the above derivation for the sake of simplicity. A corresponding derivation verifying the formula is then constructed in the next way:
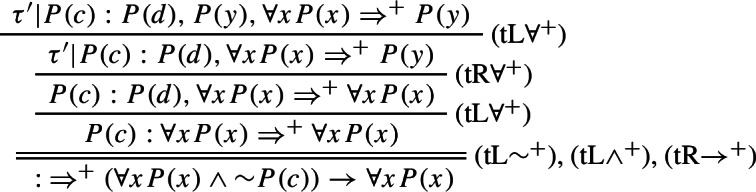
 where $$\tau '$$ is $$P(c):P(d),\forall {x}P(x)\Rightarrow ^{+}\forall {x}P(x)$$.

We shall next establish results related to the notion of balancedness for **bTKQC**. For this purpose, the classes $pos^{+}$, $pos^{-}$ etc. are re-defined as sets of predicate symbols rather than sets of atomic formulas, and clauses for quantifiers are incorporated.

### Definition 15

We have the following clauses for atomic formulas and quantifiers. 
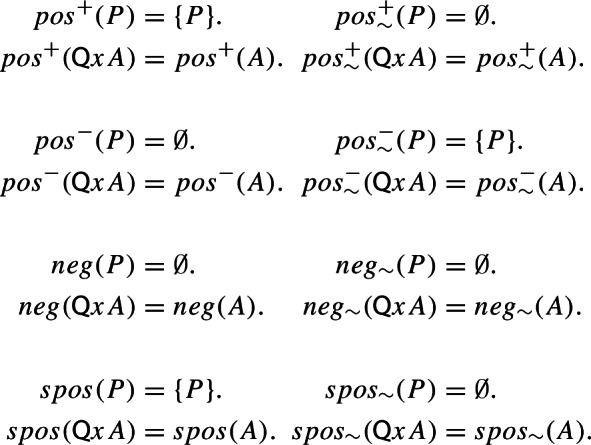
 where $$\textsf{Q}\in \{\forall ,\exists \}$$

It is easy to check that $pos^{+}(A[t_{1},\ldots ,t_{n}])=pos^{+}(A[t'_{1},\ldots ,t'_{n}])$ for any formula *A* and any terms $t_{1},\ldots ,t_{n}$ and $t'_{1},\ldots ,t'_{n}$; this applies to other classes as well.

### Proposition 40

In **bTKQC**, the conclusion of a rule is balanced only if its premise(s) are.

### Proof

By inspection of the rules. Here we consider the rules for the universal quantifier; the cases for the existential quantifier is treated analogously.

For (L$\forall ^{-}$), consider an instance:

$$\begin{aligned} \frac{\sigma |A[x/y],\Gamma :\Delta \Rightarrow ^{*}C|\tau }{\sigma |\forall {x}A,\Gamma :\Delta \Rightarrow ^{*}C|\tau } {(\text {tL}\forall ^{-})} \end{aligned}$$

Then if a sequent in $\sigma$ or $\tau$ is balanced, the same sequent can be used as a witness for the balancedness of the premise. Otherwise, we have

$$\begin{aligned} spos(C)\cup spos_{\mathord {\sim }}(C)\subseteq &   (pos^{-}_{\mathord {\sim }}(\{\forall {x}A\}\cup \Gamma )\cup pos^{+}_{\mathord {\sim }}(\Delta )\cup neg_{\mathord {\sim }}(C))\\  &   \cap (pos^{-}(\{\forall {x}A\}\cup \Gamma )\cup pos^{+}(\Delta )\cup neg(C)). \end{aligned}$$

Now since $pos^{-}_{\mathord {\sim }}(\forall {x}A)=pos^{-}_{\mathord {\sim }}(A[x/y])$ and $pos^{-}(\forall {x}A)=pos^{-}(A[x/y])$, we have:

$$\begin{aligned} spos(C)\cup spos_{\mathord {\sim }}(C)\subseteq &   (pos^{-}_{\mathord {\sim }}(\{A[x/y]\}\cup \Gamma )\cup pos^{+}_{\mathord {\sim }}(\Delta )\cup neg_{\mathord {\sim }}(C))\\  &   \cap (pos^{-}(\{A[x/y]\}\cup \Gamma )\cup pos^{+}(\Delta )\cup neg(C)). \end{aligned}$$

as required. Other cases are treated similarly.

$$\square $$

### Definition 16

For a predicate symbol *P*, we define two **QC**-models $\mathcal {M}^{P}_{1}$ and $\mathcal {M}^{P}_{2}$ in the following way: $\mathcal {M}^{P}_{1}{:}{=}(({w},\{(w,w)\}),\Delta ,\delta ,\mathcal {V}^{+}_{1},\mathcal {V}^{-}_{1})$ s.t. $\Delta$ is the set of terms of $\mathcal {L}_{q}$; $\delta (w)=\Delta$; $\mathcal {V}^{+}_{1}(Q(t_{1},\ldots ,t_{n})){:}{=}\emptyset$ if $Q=P$, and $\{w\}$ otherwise; and $\mathcal {V}^{-}_{1}(Q(t_{1},\ldots ,t_{n})){:}{=}\{w\}$. $\mathcal {M}^{P}_{2}$ is defined similarly, except that $\mathcal {V}^{-}_{2}(Q(t_{1},\ldots ,t_{n})){:}{=}\emptyset$ if $Q=P$, and $\{w\}$ otherwise; and $\mathcal {V}^{+}_{2}(Q(t_{1},\ldots ,t_{n})){:}{=}\{w\}$.

As in the propositional case, we now turn to the semantical side, in order to confirm that a provable contradiction must be balanced.

### Lemma 41

The following statements hold.

(i) If $$P\notin pos^{+}(A)$$ , then $$\mathcal {M}^{P}_{1},w\Vdash ^{+}A$$.
(ii) If $P\notin pos^{+}_{\mathord {\sim }}(A)$, then $\mathcal {M}^{P}_{2}, w\Vdash ^{+}A$.

### Proof

(i) By induction on *A*. The cases for propositional connectives are similar to those in Lemma 11.

If $A\equiv Q(t_{1},\ldots , t_{n})$, then for the case $P\equiv Q$, $P\in pos^{+}(A)$, so the statement follows. If $P\not \equiv Q$, then $\mathcal {M}^{P}_{1},w\Vdash ^{+}Q(t_{1},\ldots ,t_{n})$ by definition of $\mathcal {V}^{+}_{1}$.

If $A\equiv \forall {x}B$, then $P\notin pos^{+}(\forall {x}B)$ implies $P\notin pos^{+}(B[x/t])$ for all $t\in \delta (w)$. So by the I.H. $\mathcal {M}^{P}_{1},w\Vdash ^{+}B[x/t]$ for all $t\in \delta (w)$. Consequently $\mathcal {M}^{P}_{1},w\Vdash ^{+}\forall {x}B$.

If $A\equiv \mathord {\sim }\forall {x}B$, then $P\notin pos^{+}(\mathord {\sim }\forall {x}B)$ implies $P\notin pos^{-}(\forall {x}B)$ and so $P\notin pos^{-}(B)$. Thus $P\notin pos^{+}(\mathord {\sim }B)$ and by the I.H. $\mathcal {M}^{P}_{1},w\Vdash ^{+}\mathord {\sim }B$. Consequently $\mathcal {M}^{P}_{1},w\Vdash ^{+}\mathord {\sim }\forall {x}B$. The cases for a (negated) existential formula and (ii) are analogous.

$$\square $$

### Lemma 42

Assume $P\in spos(A)\cup spos_{\mathord {\sim }}(A)$. Then the next statements hold.

(i) If $P\notin neg(A)$, then $\mathcal {M}^{P}_{1},w\nVdash ^{+}A$ or $\mathcal {M}^{P}_{1},w\nVdash ^{-}A$.
(ii) If $P\notin neg_{\mathord {\sim }}(A)$, then $\mathcal {M}^{P}_{2},w\nVdash ^{+}A$ or $\mathcal {M}^{P}_{2},w\nVdash ^{-}A$.

### Proof

By induction on the complexity of *A*. Here we look at the case for (i). The cases for propositional connectives are analogous to Lemma 12.

If $A\equiv Q(t_{1},\ldots ,t_{n})$ then $P\in spos(Q(t_{1},\ldots , t_{n}))\cup spos_{\mathord {\sim }}(Q(t_{1},\ldots ,t_{n}))$ implies $P\equiv Q$, so $\mathcal {M}^{P}_{1},w\nVdash ^{+}Q(t_{1},\ldots , t_{n})$.

If $A\equiv \forall {x}B$, then $P\in spos(\forall {x}B)\cup spos_{\mathord {\sim }}(\forall {x}B)$ and $P\notin neg(\forall {x}B)$ implies $P\in spos(B)\cup spos_{\mathord {\sim }}(B)$ and $P\notin neg(B)$. Hence by the I.H. $\mathcal {M}^{P}_{1}\nVdash ^{+}B$ or $\mathcal {M}^{P}_{1}\nVdash ^{-}B$. Given that $\mathcal {M}^{P}_{1}$ treats all terms equally, it then follows that $\mathcal {M}^{P}_{1}\nVdash ^{+}\forall {x}B$ or $\mathcal {M}^{P}_{1}\nVdash ^{-}\forall {x}B$. The case for $\exists$ follows similarly. $\square$

We then obtain a statement analogous to Theorem 24.

### Theorem 43

If $\textbf{bTKQC}\vdash \ :\ \Rightarrow ^{+}A$ and $\textbf{bTKQC}\vdash \ :\ \Rightarrow ^{-}A$, then the tableaux are balanced.

### Proof

This follows from Lemma 41, 42.

$$\square $$

### Corollary 44

If a construction of a derivation of **bTKQC** begins with an instance of ($\text {tqAx}^{-}$) or ($\text {tqAx}^{+}$) that is not balanced, then it never derives a provable contradiction.

### Proof

This follows as in Corollary 25, using Theorem 43 and Proposition 40. $\square$

## Concluding remarks

In this paper, we studied the interaction of provable contradictions in **C** and possible correspondences in their derivations. We proposed a notion of correspondence of derivations for bilateral sequent and tableau calculi. Using this notion, we observed that for certain calculi of **C** (based on different languages), the notion of correspondence provides a necessary condition for provable contradictions. Furthermore, we made an additional analysis of the phenomenon in terms of types of propositional variables, employing a notion which we termed *balancedness*.

For future directions, it can be asked whether the analyses of this paper extend to other contradictory logics, especially in the vicinity of **C**. One such endeavour has in fact already been taken [17], in the case of *Francez-Weiss* logics, which are relevant subsystems of the ${\textbf {C}}_{\rightarrow }$. Another worthwhile direction might be to study the relationship between our notion of correspondence and the notion of *proof-theoretic synonymy* [3, 25], which offers a different criterion to measure the similarity of derivations for logics that employ strong negation. It could be of interest to see how suited the latter notion is for investigating provable contradictions, and which of the notions has a comparative advantage therein.

## Data Availability

No datasets were generated or analysed during the current study.

## Footnotes

1 The semantical perspectives of **C** and related systems are investigated in detail by D. Fazio and S.P. Odintsov [6] as well as Kripke-semantically by G.K. Olkhovikov [18].

2 This representation is philosophically favoured in *logical multilateralism* endorsed by H. Wansing and S. Ayhan [23, 26]. It is also related to the theory of *signed consequence relation* introduced by S.A. Drobyshevich [4, 5].

3 This term is here used to contrast the systems with (*unilateral*) calculi for **N4** (see again [9, 10]), which have no signs or different kinds of antecedents, and which typically employ different negation rules for each connective.

4 For other formulations of sequent calculi for **C**, see e.g. [2, 8, 11, 16, 19].

5 To be precise, invertibility does not hold for (R$\wedge ^{-}$), (R$\vee ^{+}$) as well as (L$\rightarrow ^{-}$) and (L$\rightarrow ^{+}$) with respect to the left premise.

6 That is to say, $\emptyset :\emptyset \Rightarrow ^{+}A$ and $\emptyset :\emptyset \Rightarrow ^{-}A$.

7 One may equally start from a derivation of $\ :\ \Rightarrow ^{-} A$.

8 We will come back to this notion more precisely in Section 3.

9 There is not a corresponding notion of *strictly negatively*, as the set of variables in such occurrences has to be empty. On the other hand, it is possible to collect variable occurrences that occur *non-strictly positively*; see [7] for details.

10 Note the abuse of notation here in that $\Delta \cup \{A\}$ is a multiset union while other $\cup$ are of sets. The difference is however not important in the present context.

11 The deviation in the definition of *spos* and $spos_{\mathord {\sim }}$ was needed for this step.

12 In comparison, Mint’s first-order tableaux calculus in [13] is aimed at proof-search, and more sophisticated than his propositional system.
